# Neural mechanisms of lipreading in the Polish-speaking population: effects of linguistic complexity and sex differences

**DOI:** 10.1038/s41598-025-98026-8

**Published:** 2025-04-17

**Authors:** Jakub Wojciechowski, Joanna Beck, Hanna Cygan, Agnieszka Pankowska, Tomasz Wolak

**Affiliations:** 1 Bioimaging Research Center, Institute of Physiology and Pathology of Hearing, 10 Mochnackiego St, Warsaw, 02-042 Poland; 2 Nencki Institute of Experimental Biology, Polish Academy of Sciences, 3 Pasteur St, Warsaw, 02-093 Poland; 3 Medical Faculty, Lazarski University, Warsaw, 02-662 Poland; 4 Rehabilitation Clinic, Institute of Physiology and Pathology of Hearing, 10 Mochnackiego St, Warsaw, 02-042 Poland

**Keywords:** Lipreading, Speech-reading, Audiovisual integration, Sex differences, fMRI, Language comprehension, Medical imaging, Auditory system, Cognitive neuroscience, Social neuroscience, Visual system

## Abstract

Lipreading, the ability to understand speech by observing lips and facial movements, is a vital communication skill that enhances speech comprehension in diverse contexts, such as noisy environments. This study examines the neural mechanisms underlying lipreading in the Polish-speaking population, focusing on the complexity of linguistic material and potential sex differences in lipreading ability. Cohort of 51 participants (26 females) underwent a behavioral lipreading test and an fMRI-based speech comprehension task, utilizing visual-only and audiovisual stimuli, manipulating the lexicality and grammar of linguistic materials. Results indicated that males and females did not differ significantly in objective lipreading skills, though females rated their subjective abilities higher. Neuroimaging revealed increased activation in regions associated with speech processing, such as the superior temporal cortex, when participants engaged in visual-only lipreading compared to audiovisual condition. Lexicality of visual-only material engaged distinct neural pathways, highlighting the role of motor areas in visual speech comprehension. These findings contribute to understanding the neurocognitive processes in lipreading, suggesting that visual speech perception is a multimodal process involving extensive brain regions typically associated with auditory processing. The study underscores the potential of lipreading training in rehabilitating individuals with hearing loss and informs the development of assistive technologies.

**Supplementary Information:**

The online version contains supplementary material available at 10.1038/s41598-025-98026-8.

## Introduction

Lipreading is the ability to extract speech information from the movements of a speaker’s lips and face. It is far from being a specialized skill limited to those with hearing impairments, and plays a significant role in everyday communication across the general population. It is particularly vital in environments where auditory cues are insufficient or absent, such as noisy public spaces or situations where individuals must maintain silence. Visual information from the talker’s face helps fill in the missing auditory information (e.g.^1,2^). The universality of lipreading is underscored by its inclusion in early communication development, with infants showing sensitivity to visual speech cues even before they develop full auditory speech capabilities^3^. Articulatory lip movements enable visemes recognition (the visual equivalent of phonemes) and supplement degraded auditory information during speech perception. Despite its practical importance, the neural and cognitive mechanisms underlying lipreading still need to be better understood. However, recent advances in neuroscience and psychology have shed new light on the neural networks involved in visual speech perception and the role of visual cues in speech comprehension (e.g.^4,5^).

In particular, neuroimaging studies have shown that the brain regions involved in lipreading overlap with those involved in auditory speech processing, suggesting that lipreading relies on similar neural mechanisms as normal hearing, including the auditory cortex^6,7^. Additionally, despite simplifying lipreading as “hearing without sounds”, brain regions associated with language processing, such as the left inferior frontal gyrus (IFG) and posterior superior temporal gyrus (pSTG), and visual cortex are also activated^8,9^. Furthermore audiovisual integration during lipreading showed involvement of the superior temporal sulcus and pSTG^10,11^. These regions similarly engage in auditory speech perception and comprehension in individuals with hearing impairments and among normal hearing populations^12,13^.

The contribution of visual processing during lipreading has been highlighted in recent studies. ^4^Peelle et al. (2022) conducted a brain imaging study to investigate the neural mechanisms underlying audiovisual integration processes. The researchers examined the brain activity of 60 healthy adults while they processed visual-only, auditory-only, and audiovisual words. The results revealed enhanced connectivity between the auditory, visual, and premotor cortex during audiovisual speech processing compared to unimodal processing. Furthermore, during visual-only speech processing, there was increased connectivity between the posterior superior temporal sulcus (pSTS) and the primary visual cortex (V1), but not the primary auditory cortex (A1), across most experimental conditions. The authors proposed that the pSTS region might be crucial in integrating visual information with an existing auditory-based perception. These findings supported the earlier research by ^14^Zhu and Beauchamp (2017), who found that different regions of the pSTS preferred visually presented faces with either moving mouths or moving eyes, with only the mouth-preferring regions exhibiting a strong response to voices. However, what remains unclear and continues to be debated is the involvement of the premotor cortex in speech perception across various paradigms, particularly in terms of lexicality and the modality of the stimulus^4,15^.

Moreover, the presence of visual-related responses in the superior temporal cortex (STC) of individuals who are deaf may be attributed to long-term auditory deprivation, such as the absence of auditory sensory input. However, it could also be influenced by other dynamic cognitive functions, such as the acquisition of sign language^12^. Previous research has shown that the activity in the STC positively correlates with the duration of deafness or the age at which cochlear implants were received^16–19^ indicating that functional reorganization likely occurs in the auditory cortex over an extended period. Systematic review and meta-analysis which discusses how increased activation in the STC in response to visual speech leads to improved speech understanding revealed that STC activation corresponds to the ability to read lips and understand speech rather than the duration of sensory deprivation^20,21^. This suggests that the compensatory changes resulting from sensory deprivation do not necessarily require a gradual integration of visual inputs into the STC. Instead, they are rapidly modulated by preexisting connections from higher-level cortical areas associated with language processing. Hence, the reorganization of the STC may involve contributions from both bottom-up signals (e.g., originating from the visual cortex) and top-down modulation (e.g., originating from the frontal-temporal regions) to facilitate such cross-modal activity^22^.

Research has provided evidence that extended training in lipreading can bring about structural and functional changes in the brain regions involved in visual and auditory processing among proficient lip readers^9,23,24^. Furthermore, studies have demonstrated neuroplasticity related to lipreading in deaf individuals, who heavily rely on lipreading, and exhibit heightened visual processing in brain areas typically associated with auditory processing^25,26^. These findings contribute to our understanding of how lipreading supports speech perception and have potential implications for rehabilitation strategies and the development of assistive technologies for individuals with hearing impairments.

Previously, audiovisual integration was often regarded as an “individual difference” variable, unrelated to unimodal processing abilities^27,28^. However, ^29^Tye-Murray et al. (2016) demonstrated that word recognition scores for auditory-only and visual-only stimuli accurately predicted audiovisual speech perception performance with no evidence of a distinct integrative ability factor. These findings may suggest that audiovisual speech perception relies primarily on coordinating auditory and visual inputs. In summary, while significant insights have been gained into the neural mechanisms of lipreading and its overlap with auditory speech processing, the specific involvement of the premotor cortex and how it varies by lexicality and stimulus modality during lipreading remains poorly understood and debated.

What is more, gender appears to play an important role in lipreading, although findings on sex differences have been inconsistent. Some studies suggest that women outperform men in this skill. For instance^30^, found that women performed better than men in a lipreading task requiring participants to identify speech sounds solely from visual cues^31^ reported higher lipreading accuracy for women when identifying sentences from visual cues alone. However, other studies have found no significant differences in lipreading accuracy between men and women^32^. In terms of neural mechanisms, there is evidence that women and men may engage different neural pathways during lipreading. For example^33,34^, found that females exhibited greater activity in the left auditory area while lipreading silently articulated numbers, despite similar recognition accuracy to males. This suggests potential sex-based differences in neural processing, even in the absence of behavioral differences. Overall, the literature is inconsistent, leaving the nature and causes of these differences unclear. To account for this variability, some studies have chosen to focus exclusively on one sex—predominantly females—to minimize between-sex variability (e.g.)^35^.

Behavioral studies provide a valuable framework for gaining a deeper understanding of neurobiological findings. The context in which words and sentences are presented plays a significant role in lipreading accuracy. Compared to isolated sentences, lipreading accuracy is enhanced when sentences are presented within a meaningful context, such as a story^1,36^. This means that lipreading relies on visual cues from the speaker’s lips as well as contextual information. Factors such as the visibility of the speaker’s face and the distinctiveness of lips movements also influence lipreading accuracy^37^.

Furthermore, linguistic factors, including the complexity of words and sentences, can impact lipreading accuracy^38^. Research has demonstrated a connection between lipreading ability and auditory perception, where individuals with better lipreading skills tend to exhibit superior auditory perception skills, particularly in noisy environments^10,39^. This relationship appears to stem from the integration of audiovisual information rather than reliance on one modality over the other. Studies such as^23^ suggest that shared cognitive mechanisms like attention and memory support both lipreading and auditory perception, enhancing speech comprehension in noisy settings. Furthermore^29^, showed that performance on auditory-only and visual-only tasks independently predicted audiovisual speech perception, indicating that lipreading complements rather than substitutes auditory processing. These findings highlight the dynamic interplay between modalities, wherein lipreading may augment auditory perception even in less challenging conditions, as demonstrated by the McGurk effect^27^.

Lipreading and auditory perception are intertwined and rely on shared cognitive processes such as: attention, memory, integration of multisensory information, and language processing. Importantly, training programs focusing on visual speech perception have been shown to enhance lipreading skills^12,40^, highlighting the potential for improvement in this domain. These findings underscore the potential of lipreading training for rehabilitating individuals with hearing loss or speech perception difficulties. Firstly, however, it is essential to gain a deeper understanding of the neurocognitive processes underlying this phenomenon, as well as the task-dependent and subject-dependent variability.

Building upon these identified gaps in the literature, this study aims to elucidate the neural mechanisms underlying lipreading within the Polish-speaking population, with a focus on distinguishing between visual-only and audiovisual speech processing modalities. Our primary objective was to explore how the complexity of linguistic material influences the neural processing of lipreading, and how these processes differ when both auditory and visual cues are present versus when only visual cues are available. We expected that for both audiovisual and only for visual (lipreading condition) we would observe differences in brain regions involved in grammatical processing. The anterior temporal lobe (ATL) houses a lexicon of objects and events, vital for identifying and abstracting common elements into new concepts. These concepts, such as “green leaf,” illustrate ATL’s role in semantic processing and conceptual integration. At the same time, the posterior parietal cortex (PPC) serves as a critical hub for integrating sensory information and coordinating attentional resources during speech processing and oral reading. For lipreading conditions we also assumed involvement of premotor cortex (PMv) as it plays a crucial role in planning and executing the motor movements necessary for articulating speech. It coordinates with areas like the posterior frontal eye fields (pFEF) and FEF, which are involved in controlling visual attention and eye movements, respectively, during the visual processing of speech-related cues^41^. What is more, we sought to examine potential differences in lipreading ability and neural activation patterns between male and female participants, thereby contributing to the understanding of sex-specific cognitive processing in multimodal and unimodal communication contexts. We hypothesized that women would outperform men in lipreading skills, both on subjective and objective measures. Furthermore, we anticipated that women would exhibit a more specialized pattern of brain activation during the lipreading condition, specifically in STC.

## Methodology

### Participants

Participants were recruited through social media. Out of 55 recruited participants, three were trained and practicing language therapists, and one participant did not pay attention to the tasks at hand, and therefore were excluded from further analysis. After exclusion, the sample consisted of 26 females and 25 males, aged 25.51 ± 6.55. All participants were native Polish speakers, right-handed and reported normal hearing and normal or corrected to normal (with contact lenses) vision and no psychiatric or neurological disabilities.

All participants signed informed consent forms and received monetary compensation for their time. The study was approved by the research ethics committee of Nicolaus Copernicus University in Toruń and was conducted following the Helsinki Declaration for Human Studies.

**Table 1:** Participants’ demographic and lipreading-related assessment.

Variable	Female (*N* = 26)	Male (*N* = 25)	Statistics
Age	29.25	27.85	t = 0.77, *p* =.447
Objective skill score	3.29	2.74	t = 0.59, *p* =.556
Subjective skill score for visual lexical	4.31	3.26	t = 3.38, *p* =.001
Subjective skill score for visual non-lexical	1.52	1.45	t = 0.49, *p* =.627

### Lipreading comprehension test

Initially, participants watched a short video clip with sound, featuring an actress (trained speech therapist specializing in working with the hearing impaired) narrating a 20-second story. This served to acquaint them with the actress’s speech characteristics, such as speech rate and tone. Subsequently, we assessed each participant’s lipreading ability through a behavioral task conducted before the fMRI examination. Afterwards, participants viewed a different, silent, 44-second video clip of the same actress narrating a story on a specific topic (food), which was known to them in advance. After watching the video, participants were provided with a printed list of words and asked to identify those spoken by the actress. Points were awarded for correctly marked words and deducted for incorrect ones. The highest achievable score was 21, while the lowest was − 21.

**Fig. 1:**
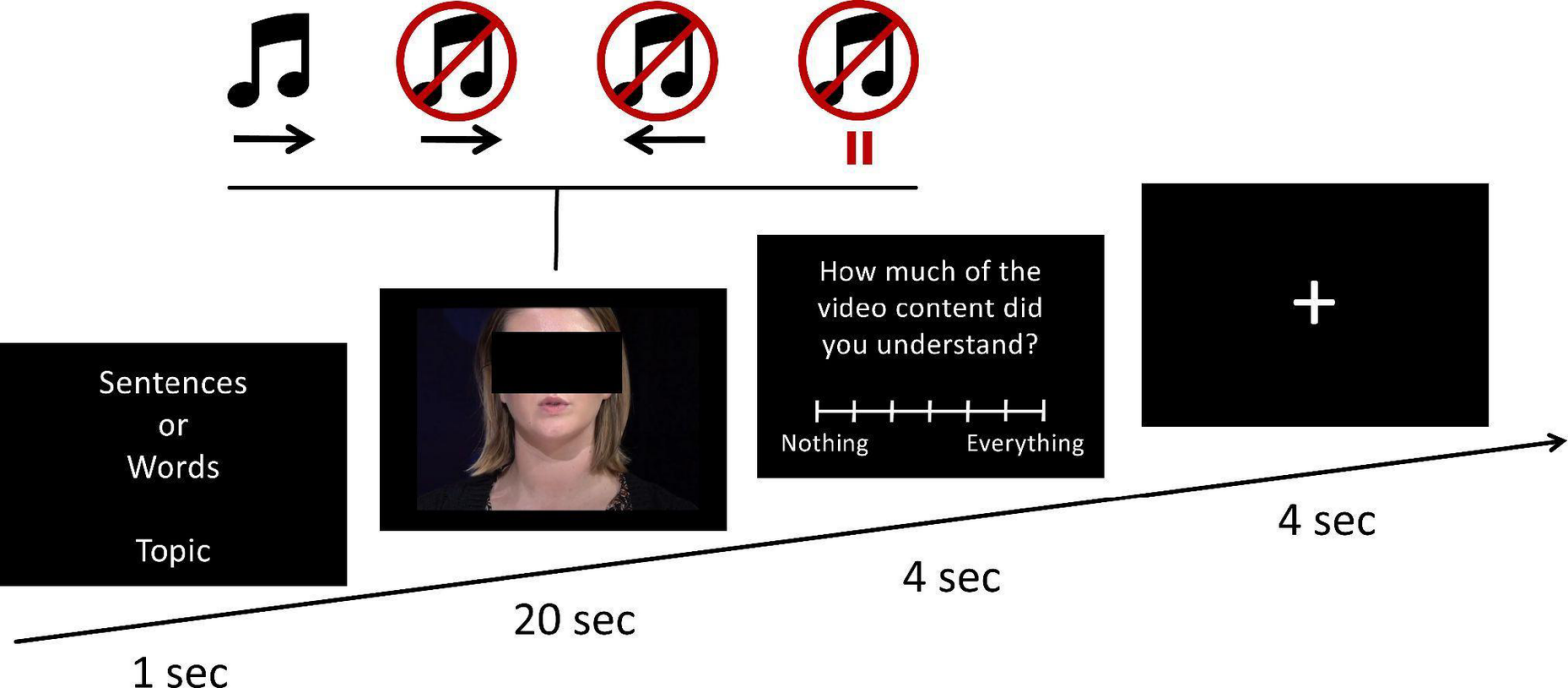
The lipreading task design. Each trial started with instruction whether full sentences or string of words will be presented and the topic of the material (e.g., sport). Following the instruction, 20 s of speech material was presented in one of three variants: (1) clip with sound (audiovisual lexical), **(2)** without sound (visual lexical), (3) without sound and played backwards (non-lexical visual). Additionally, 25% of the time, a static face of the voice actress without speech material (static-control) was presented instead. After the speech material, an interactive scale was presented. Participants were instructed to indicate how well they understood the speech material. Fixation cross was presented for 4 s after each trial.

Additionally, after each lipreading trial during the fMRI procedure, participant subjectively rated how much she/he understood from the lipreading video, by choosing a score on the 7-point Likert scale (see Fig. 1).

### Lipreading fMRI procedure

During fMRI acquisition participants performed a lipreading task. The task consisted of various visual and audiovisual materials spoken by the actress and a subsequent question about the comprehension of each material. To explore the brain’s processing of visual lexical stimuli, we used three experimental conditions. These conditions included materials spoken by the same actress: (1) naturally together with sound (audiovisual lexical); (2) naturally, but without sound (visual lexical); (3) a clip played backwards and without sound (visual non-lexical). In addition, to investigate the role of the type of linguistic material on the lexical processing of visual stimuli, each of the above conditions was implemented in the form of either narrative sentences or strings of words. The narrative sentences had simple grammatical construction and were related to everyday life. All the words were nouns, and were selected from the set of nouns occurring in the narrative sentences. Sample experimental stimuli are available online. As a control condition, we used a still photo of the voice actress with no acoustic stimulation. Consequently, we used six experimental conditions and one control condition. Each trial of the task began with information (1 s) about the topic of upcoming language material and whether it would be sentences or words. Then a clip of the language material was displayed (20 s) in line with the described experimental conditions. After the clip ended, a 7-point scale (4 s) appeared allowing participants to indicate their comprehension level of the presented language material. Each trial ended with a fixation cross (4 s). Participants subjectively rated their comprehension of the presented material using a response pad held in their right hand. Task was divided into six parts, lasting 4:03 min each, to conform with optimal fMRI sequence length. In order to avoid participants’ confusion and for more robust fMRI data modeling, each of the six task parts had only either words or sentences in an alternating order. The first part always had only sentences, and the second only words, and so on. The order of conditions inside each part of the task was semi-randomised to avoid the same condition occurring twice in a row. Materials were related to: sport, weather, food or fashion.

The experimental protocol was controlled by the Presentation software (Neurobehavioral Systems Inc.) The stimuli were presented via a mirror mounted to the MR coil and displayed on a LCD screen (NordicNeuroLab AS) inside the MRI room. Behavioral responses were collected using MR-compatible response pads (SmitsLab).

### MRI acquisition

Neuroimaging was performed using a 3 T Siemens Prisma MRI scanner equipped with a 20-channel phased-array RF head coil. Functional data for all tasks were acquired using a multi-band echo-planar-imaging sequence (TR = 1500 ms, TE = 27 ms, flip angle = 90°, FOV = 192 × 192 mm, 94 × 94 mm image matrix, 48 transversal slices of 2.4 mm slice thickness, voxel size of 2.0 × 2.0 × 2.4 mm, Slice Accel. Factor = 2, In-Plane Accel. Factor = 2, IPAT = 4, TA = 4:03 min per run). Structural images were collected with a T1-weighted 3D MP-Rage sequence (TR = 2300 ms, TE = 2.26 ms, TI = 900 ms, 8° fip angle, FOV = 208 × 230 mm, image matrix 232 × 256 mm, voxel size of 0.9 × 0.9 × 0.9 mm, 208 slices of 0.90 mm slice thickness, TA = 4:53 min).

### Behavioral analysis

To test whether males and females differ in terms of lipreading skills, we ran the t-Student tests to compare objective lipreading comprehension before neuroimaging as well as on the subjective comprehension levels during the main lipreading task. Additionally, we run Pearson correlations to check the relation between subjective and objective skill. All analysis was conducted and plotted using R^42^ with cut-off at p-value 0.05. All scripts and data used for behavioral analysis are available here: https://osf.io/6k74t/.

### Neuroimaging data preprocessing and analysis

Neuroimaging data was preprocessed using SPM12^43^. Functional data was spatially realigned to the mean image, to which the structural image was then co-registered. Segmentation and normalization to the common MNI space was performed based on high-resolution structural images with resampling to 1 mm isometric voxels. The obtained transformation parameters were applied to the functional volumes with resampling to 2 mm isometric voxels. The normalized functional images were spatially smoothed with a Gaussian kernel of full-width half-maximum (FWHM) of 6 mm, and 0.004 Hz high-pass filtered (time constant of 256 s).

Statistical modeling of fMRI data was performed in SPM12 using a general linear model. The period of speech material (20 s) was modeled for each condition type, resulting in four regressors of interest (bi-modal, lipreading lexical, lipreading non-lexical, static-control) per fMRI run, since in every run only either words or sentences were presented. As a consequence, the first run had four regressors related to words, the second run had four regressors related to sentences and so on in an alternating fashion. Additionally, six head movement parameters obtained from the realignment procedure were added to the model as nuance regressors for each run. For each participant, contrasts between estimated parameters (β values) of conditions were performed by subtraction.

For second-level group analysis, we performed a series of one-sample t-tests on the contrasts estimated parameters. We conducted analyses in three domains. First, we tested the effects of lexical lipreading processing separately for sentences and words. Second, we compared brain responses during lipreading of sentences and words, separately for lexical lipreading and non-lexical lipreading conditions. Third, based on previous literature highlighting possible sex differences in lipreading ability, we compared all the above contrasts between all male and female participants using a series of two-sample t-tests. All neuroimaging figures were plotted using BrainNetView toolbox^44^.

## Results

### Behavioral results

**Fig. 2:**
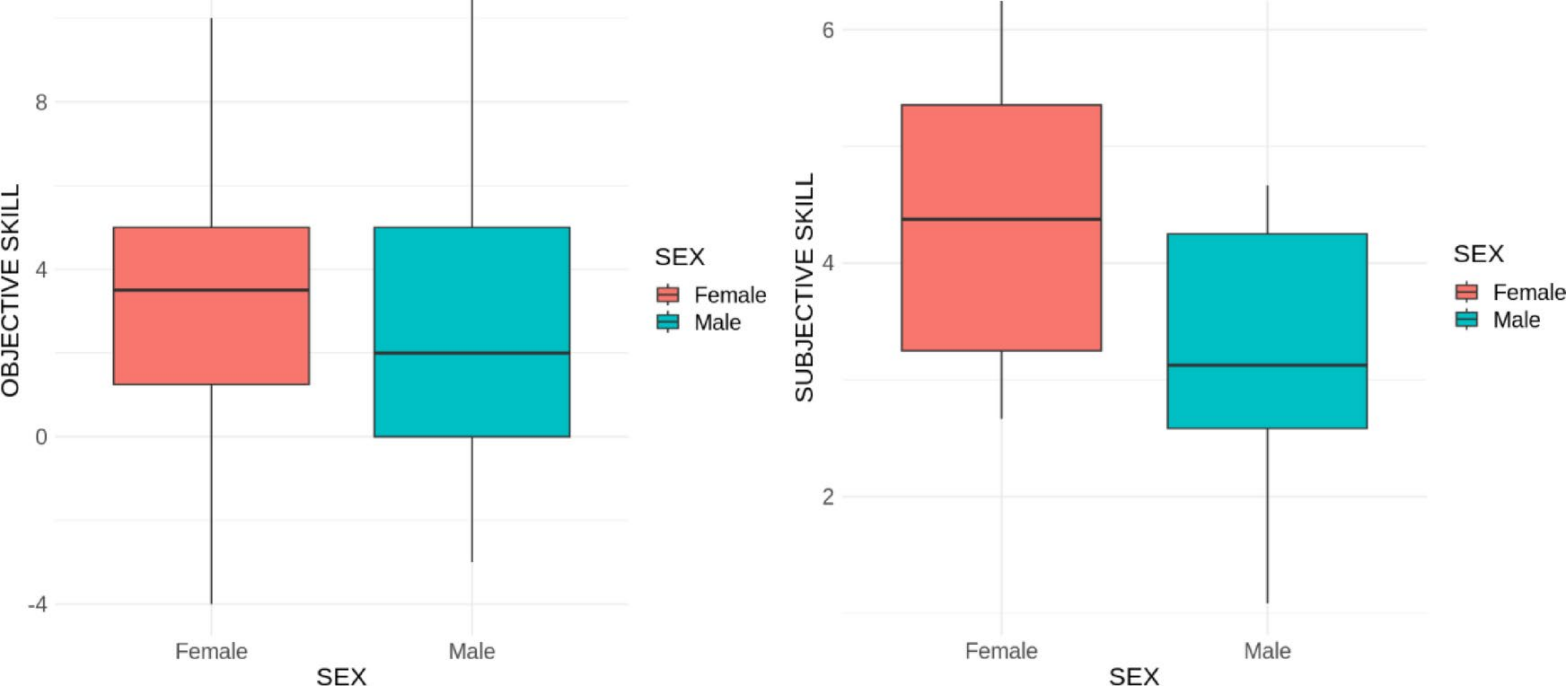
Sex differences for subjective and objective lipreading comprehension levels.

Results showed that males and females did not differ in terms of objective lipreading skill, but they did differ in terms of subjective lexical lipreading skill - females judge their lipreading comprehension level higher than males (see Table 1; Fig. 2). All the statistics and means for objective and subjective skill are listed in Table 1.

**Fig. 3:**
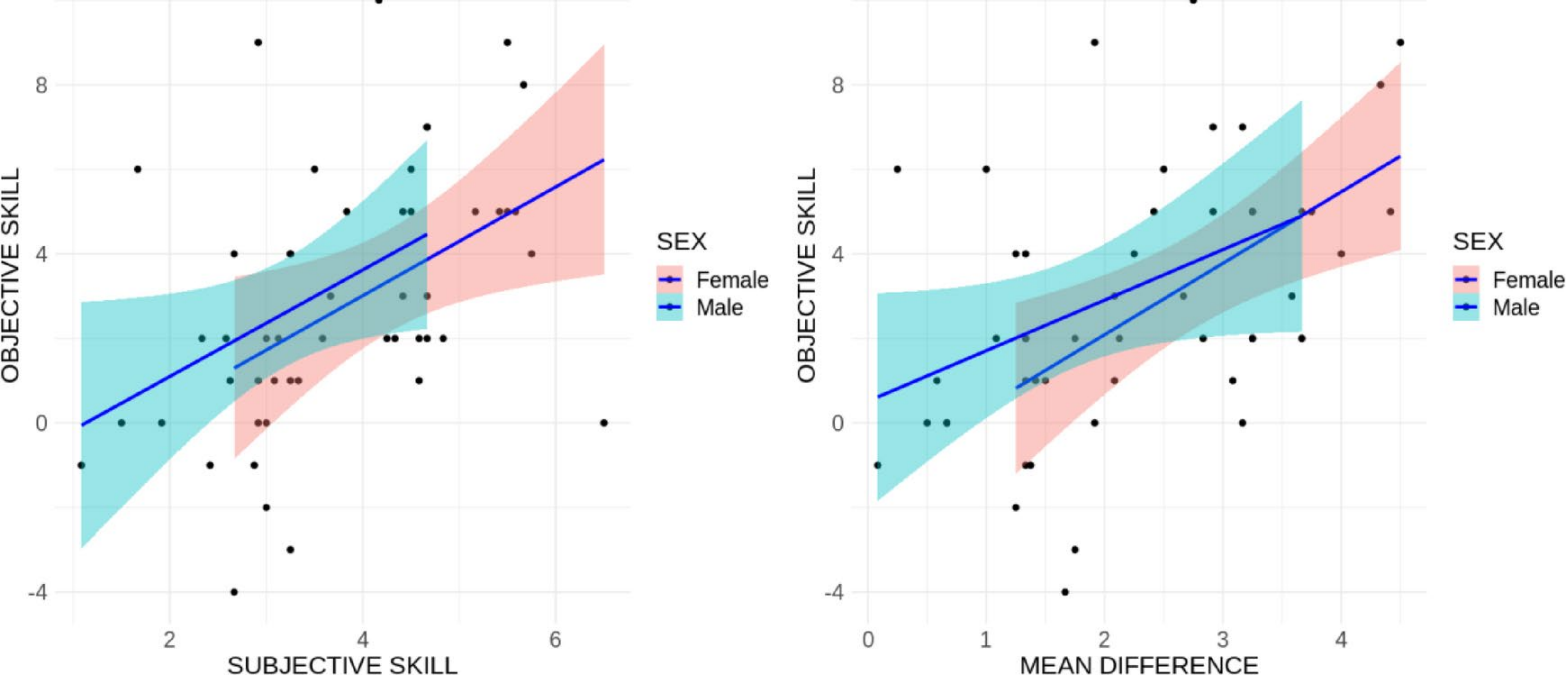
Correlation between objective lipreading skill and subjective lexical lipreading comprehension levels, and difference for lexical vs. non-lexical lipreading comprehension levels.

Additionally, objective lipreading comprehension levels was positively correlated, both for females and males (*r* =.43; *p* <.001) as well as the difference for lexical vs. non-lexical lipreading comprehension levels (*r* =.47; *p* <.001; see Fig. 3).

### Neuroimaging results

Note that in the main texts of the manuscript we do not report tables with voxel-wise statistics. They are reported in the supplementary materials. Additionally, all of the reported results are available as unthresholded maps at the neurovault repository. Figures with regions involved in audiovisual words and sentences processing (Figure S1) and conjunction analysis for ‘visual lexical vs. face’ with ‘audiovisual lexical vs. face (Figure S2) can also be found in supplementary materials.

### Sentences conditions

**Fig. 4:**
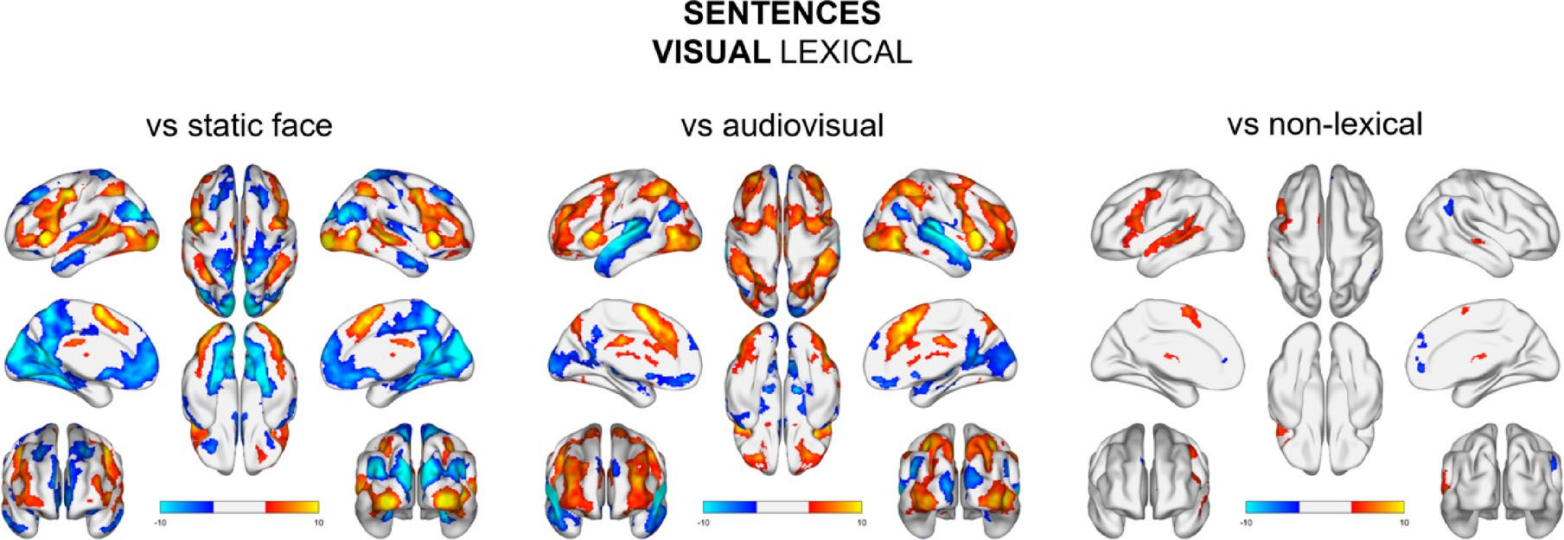
Brain map activations for visual lexical sentences comparisons to static face condition (left), lexical audiovisual condition (middle) and visual non-lexical condition. Contrast maps are thresholded at voxel-level *p* <.001 and FWE‐corrected (*p* <.05) for cluster size.

When examining the processing of visual lexical sentences in comparison to static face images, increased activation in areas associated with speech processing was noted, such as the bilateral middle and superior temporal cortex. Additionally, stronger activity was observed in the bilateral frontal and middle superior frontal areas, which encompasses supplementary motor areas (SMA). Furthermore, bilateral occipital cortex and bilateral caudate also exhibited heightened activation in response to lexical sentence processing. Results from these contrasts should be interpreted as control results, reflecting the sensitivity of our paradigm (Fig. 4, Table S7 & S8).

When we compared activation during speech processing of audiovisual and visual lexical sentences, we found that there was higher activation for audiovisual sentences in bilateral temporal and parietal areas. In bilateral frontal, parietal (cuneus, PPC) and occipital areas we observed opposite pattern, i.e. higher activation for visual lexical (Fig. 4, Table S9 & S10).

Additionally, we checked which regions were involved in visual lexical processing during sentence reading in comparison to non-lexical stimuli. We found that there were differences in bilateral superior and middle temporal gyrus (notably smaller in the right hemisphere) and in the left supplementary motor area. Visual non-lexical sentences activated the right hemisphere more strongly and involved STG/planum temporale (PT) and medial dorsolateral prefrontal cortex (Fig. 4, Table S11& S12).

### Words conditions

**Fig. 5:**
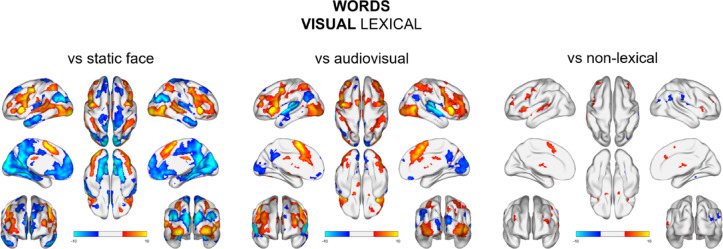
Brain map activations for lexical visual words comparisons to static face condition (left), lexical audiovisual condition (middle) and visual non-lexical condition (right). Contrast maps are thresholded at voxel-level *p* <.001 and FWE‐corrected (*p* <.05) for cluster size.

For words, as with sentences, we checked which regions are involved in visual lexical word processing in comparison to static face image. Similarly, we found that there was higher activation in speech-related areas, i.e., bilateral middle and superior temporal cortex, bilateral frontal and middle superior frontal areas (i.e. SMA), bilateral occipital cortex and bilateral caudate. Those results should be interpreted as control results, reflecting the sensitivity of our paradigm (Fig. 5, Table S13 & S14).

For visual compared to audiovisual lexical word processing, we observed higher activation in the bilateral inferior and middle frontal, bilateral inferior and superior parietal and bilateral middle and inferior occipital areas. Whereas for audiovisual word, we observed higher activation in bilateral superior and middle temporal areas, bilateral middle superior frontal gyrus, bilateral precuneus, bilateral lingual gyrus and superior occipital gyrus (Fig. 5, Table S15 & 16).

Lastly, we checked which areas were involved in the visual lexical word processing (vs. non-lexical words) and we found areas of language network, i.e., bilateral SMA, bilateral middle frontal areas and left superior and IFG, left middle and STG and left precentral gyrus. Similarly, to sentences, non-lexical words activated more the right STG/PT and middle occipital gyrus (MOG), supramarginal and angular gyri with a small cluster in fusiform gyrus (Fig. 5, Table S17 & 18).

### Visual conditions

**Fig. 6:**
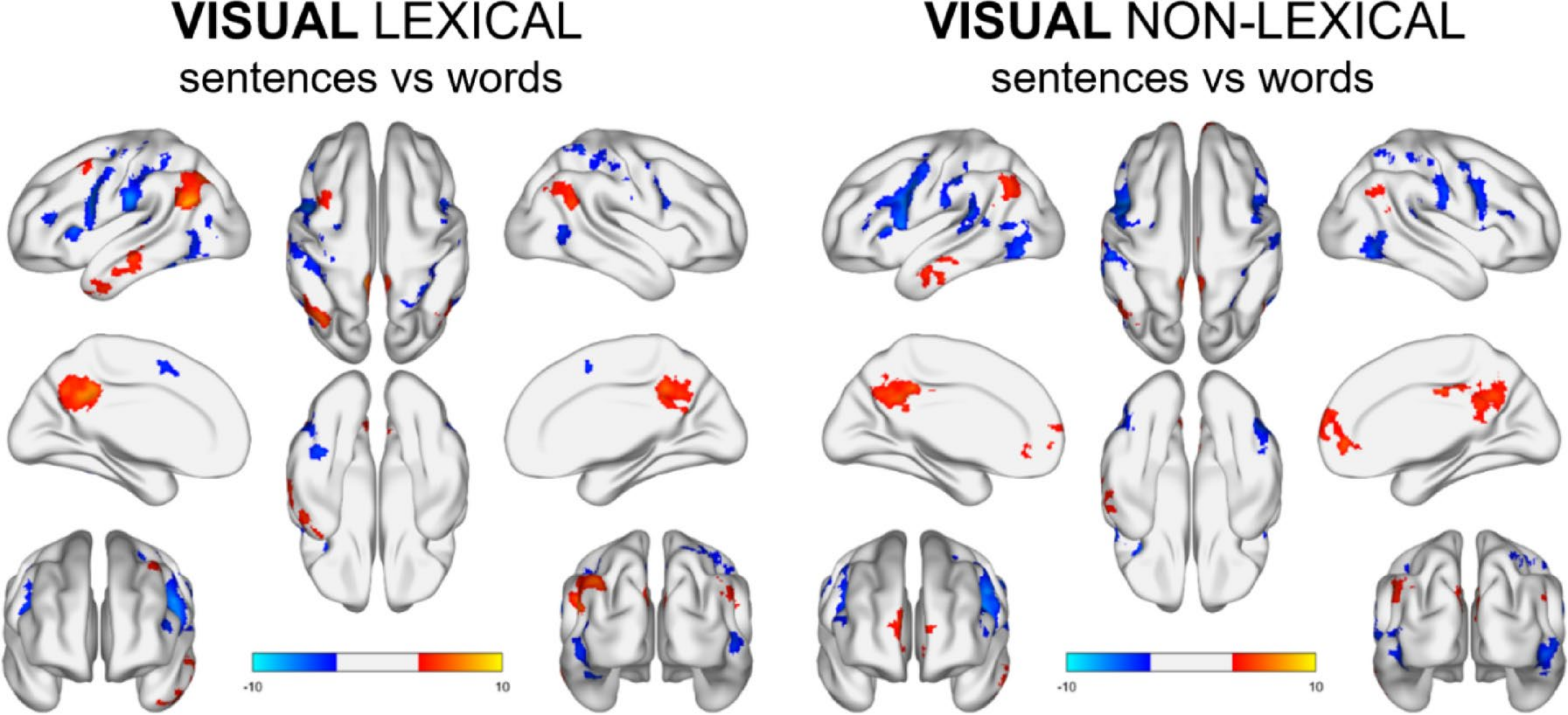
Brain map activations for visual lexical sentences vs. words comparisons (left) and visual lexical words vs. sentences comparisons (right). Contrast maps are thresholded at voxel-level *p* <.001 and FWE‐corrected (*p* <.05) for cluster size.

Comparing brain activations during processing of visual lipreading of words and sentences, we observed higher activation for sentences in bilateral precuneus, bilateral cingulate gyrus, bilateral middle frontal gyrus and left inferior temporal gyrus. On the other hand, for visual words processing, we observed heightened activation in bilateral occipital areas (including left fusiform), bilateral IFG, right cerebellum, right pre- and post-central gyrus, and left STG (Fig. 6, Table S3 & S4). Comparing the brain activity during processing of audiovisual of words and sentences, we observed more extensive differences but in the same areas as in the visual lexical condition (see: Supplementary materials).

For similar comparison but without lexical meaning, we observed differences in the same areas as in lexical, but without bilateral medial superior frontal gyrus (Fig. 6, Table S5 & S6).

Additionally, comparing visual lexical sentences vs. lexical words to visual non-lexical sentences vs. non-lexical words, one cluster of activity difference was observed in the anterior cingulate cortex with the peak in x = 4, y = 30, z = 34, voxels = 158, t = −5.01.

As evident from our observations, the activation maps for both lexical and non-lexical comparisons displayed notable similarities.

### Sex differences

We observed no differences in brain activity between males and females for any of the contrasts of the lipreading conditions.

## Discussion

The aim of this study was to investigate the neural underpinnings of visual speech processing during lipreading. To achieve this, we designed an fMRI-based speech comprehension task to examine three key aspects of speech processing: (1) varying levels of semantic involvement (words vs. sentences), (2) lexicality of the speech material (regular vs. backward-played), and (3) the modality of speech perception (with vs. without auditory input). Our primary objective was to explore the neural mechanisms underlying lipreading, focusing on specific regions including the anterior temporal lobe (ATL), posterior parietal cortex (PPC), and premotor cortex (PMC). We hypothesized that these regions would show significant activity during visual-only and audiovisual speech processing, with the ATL and PPC associated with linguistic complexity and the PMC engaged during visual lexical processing. Furthermore, we hypothesized heightened activity in the superior temporal cortex (STC) for female participants, reflecting potential sex-based differences in neural processing. Below, we detail how the observed results aligned with these expectations.

Neuronal activity patterns in both lexical and non-lexical comparisons during processing of words and sentences showed some similarities in activation patterns. In turn, the differentiating patterns suggest that non-lexical stimuli do not activate (or activate less) frontal and temporal areas of language networks (Figs. 4 and 5). A left-lateralized activation pattern observed in the SFG and IFG, particularly the Broca’s area, reinforces its significance in word processing, even in the absence of auditory input. Differences (enhanced activity) in superior temporal sulcus and middle temporal gyrus (MTG) related to facial expression during lexical lipreading suggest that participants were actively engaged in phoneme and lexical encoding and also involved in the retrieval of the semantic lexicon in line with:^14^.

Interestingly both variants of visual non-lexical stimuli - words and sentences, when compared to visual lexical stimuli, elicited enhanced activation in the right hemisphere in the STG. The voiceless speech played backward (non-lexical) consisted of detectable atypical eye gaze and speech-like lip movements that did not match the expected linguistic code. Non-coherent and unexpected lip and eye movements may have triggered right pSTS activity, known for its role in eye-gaze and facial expression comprehension^45^ and face-voice integration^46^ during communication. This interpretation is also supported by the involvement of the medial dorsolateral prefrontal cortex in response to non-lexical sentences. These regions, known for their engagement in various cognitive functions, including working memory and lexical retrieval^47^, appear to contribute significantly to the complex set of processes involved in speech recognition and non-verbal communication interpretation.

For visual lexical words, the involvement of the SMA, related to coordination of speech-related motor movements, has been consistently implicated in language-related tasks^48,49^. A growing body of clinical neurosurgical and neuropsychological data confirms the central role of SMA in speech production, including initiation, automatization, and monitoring of motor execution. White matter tracts of degeneration connecting the SMA to relevant cortical areas underlie symptoms of progressive apraxia and aphasia^50^. On the other hand, clinical dysfunction of SMA does not affect language comprehension^51^. Although our initial hypothesis focused on the PMC, the observed activation in the SMA aligns with our broader expectation that motor regions are involved in visual speech processing. The SMA, as part of the motor network, may play a complementary or overlapping role with the PMC in coordinating speech-related movements and analyzing visemes during lipreading.

Our findings suggest that motor aspects of speech may be especially important in visual speech comprehension than in audiovisual speech comprehension. This seems to be particularly true for the task where visually presented words appear in isolation and speech movements can be easily observed and analyzed via executive motor nodes. This was not the case in visual sentence comprehension, during which it was more difficult to extract and analyze visemes via the executive motor system and, consequently, lipreading was less effective.

When sentences and words were processed without voice, there were still observable differences in brain activation, though they were less extensive than with voice (Fig. 6, Figure S1). The effects found in the anterior and inferior temporal poles indicate a differential role for semantic information retrieval^52^ in reading words and sentences, likely due to the complexity and difficulty of the linguistic material. Additionally, ATL plays a central role in integrating semantic and syntactic information and is particularly sensitive to meaningful sentences^53^. The observed activation of the ATL during visual sentence processing aligns with our hypothesis, supporting its role in semantic integration and syntactic processing. In contrast, the temporoparietal junction’s differential involvement in reading words and sentences may be due to the high cognitive demands during sentence recognition and the involvement of extensive attentional resources in analyzing lip movements.

Modality plays a crucial role in brain activation during language recognition. However, for without voice conditions we observed stronger activation in the temporal, occipital and frontal areas than to static face condition and in occipital and frontal in comparison to with voice condition. The role of the visual system in lipreading is significant from an early stage of processing^54^. As ^55^Paulesu et al. (2003) summed up, the study by ^56^Campbell (1997) focused on patient L.M., who had bilateral lesions in the middle temporal area/V5 area. This area, identified by ^57^Zeki et al. (1991), plays a crucial role in visual motion perception. L.M. exhibited a significant impairment in lipreading and was notably less susceptible to the fusion illusion. The visual modality-specific representations of speech have been supported by further studies for review, see^58^. Recent research highlights the existence of a visuo-phonological mapping process during lipreading, which is additionally supported by complex input from motor and premotor cortices and posterior temporal areas^59^. These findings collectively suggest that the phonological analysis of speech is a multimodal process, incorporating both visual cues (such as lip movements) and auditory information. This supports the notion that speech perception involves the integration of visual and auditory elements in line with^55^.

From the results regarding brain activation during processing with and without auditory input, as we expected, we posit that modality plays a pivotal role in brain activation during language recognition. Furthermore, when information from all required inputs (auditory in this study) is lacking, the involvement of language-related regions is stronger and covers larger areas, possibly reflecting increased processing effort. Indeed, higher language ability has been associated with both increases and decreases in neural activity, with considerable variation regarding the brain areas involved. Additionally, a range of interpretations has been proposed to explain these findings^60^. Increased activity in areas of the cortical language network, such as the left angular gyrus, Broca’s area, and the left temporal lobe, has been hypothesized to reflect deeper semantic processing and greater sensitivity to semantic relationships between sentences during comprehension tasks^61,62^. A similar effect can be found when comparing brain activity during the comprehension of texts on familiar versus unfamiliar topics, which could also be explained by deeper semantic processing of familiar than unfamiliar content^63,64^. Negative relationships between brain activity and language ability have typically been interpreted as neural efficiency^65^. This concept is characterized by reduced brain activity in individuals with higher ability compared to those with lower ability, despite equal or superior performance^61^. Other researchers have suggested automatization processes to explain reduced neural activity in subjects with high language ability, as skilled readers engage in more automated and efficient processing^66^. The neural engagement observed in response to various semantic stimuli, involving key areas such as IFG/Broca, ATL, pSTS, pMTG, and the left STG, underscores the significance of considering visual speech reception as an influential processing modality involved in language comprehension. This insight contributes to a more comprehensive understanding of how linguistic information is perceived and interpreted in the brain.

Our results also added one more puzzle to the discussion about sex differences in lipreading skill and its brain mechanisms. We did not find any significant differences on behavioral and neurobiological level, which is in contrast to:^30^ and in line with^31^ or^67^. While null results do not point to a lack of effect, in current study more recent neuroimaging acquisition and processing techniques were used as well as the sample size was larger than those in previous fMRI studies. It is therefore likely that the effects of sex differences in neural processing of speech reading are small. Moreover, there exists conflicting information regarding sex differences in visual speech comprehension, likely stemming from the diverse range of protocols employed. These protocols have varied from syllable-based assessments to tests involving words and sentence comprehension^68^. In this study, we explored straightforward words and sentences. Aligning with the hypothesis that women excel in speech-reading continuous speech fragments, we anticipated that as task demands increased, sex differences would become more apparent^69^. Although we hypothesized heightened activity in the STC for female participants, no significant differences were observed, suggesting that sex-related effects in neural processing of lipreading may be subtle or influenced by task complexity.

However, our behavioral results showed that while males and females do not differ in objective lipreading skills, they do report differences in subjective assessments of these skills. Cultural and societal expectations may influence individuals’ self-perception of their lipreading abilities. Stereotypes about sex roles and communication skills might lead females to perceive themselves as more adept at tasks like lipreading, even when objective measures do not support this distinction^70,71^. Additionally, differences in communication styles or preferences between sexes might explain why females feel more comfortable or effective in certain communication tasks, such as lipreading, despite the lack of significant objective differences. It is important to note that these interpretations are speculative. The observed differences might also stem from the varying complexity of the tasks evaluated. In the fMRI study, simpler sentences and words were used, which may suggest that females generally perform better with simpler material (in line^71^. Conversely, more complex tasks, like the objective measures involving longer narratives, might pose greater challenges, potentially explaining the lack of significant differences in performance on these tasks. This aspect warrants further investigation to understand the underlying factors more comprehensively.

## Conclusions

Our results revealed key cortical areas involved in visual speech processing. Modality plays a pivotal role in language recognition, influencing neural engagement. We observed that the absence of auditory input led to enhanced activation of language-related brain regions, indicating a heightened processing effort when relying solely on visual cues. Notably, key areas such as the IFG/Broca, ATL, pSTS, pMTG, and the left middle and STG were actively engaged, underscoring the importance of visual speech reception as a significant modality in language comprehension. The visual system’s significant role in lipreading, as a multimodal process, was emphasized.

our findings also contribute to the discussion on sex differences in lipreading skill, finding no significant differences on behavioral and neurobiological levels, challenging previous research suggesting such differences. Subjective reading comprehension level varied between sexes, and perceived differences in lipreading ability may be more related to cultural and societal influences rather than inherent neurological distinctions. Overall, our study provides new insights into the neural mechanisms underlying visual-only lipreading and audiovisual language perception and sheds light on the functional differences between these two modes of speech perception. These findings may have important implications for hearing loss rehabilitation, speech recognition technologies, and cross-linguistic communication. They highlight the need for further research to better understand the neural and cognitive bases of lipreading.

In conclusion, our findings shed light on neural processes in language comprehension, emphasizing modality, voice impact, and cultural influences. Implications include understanding language disorders, brain function, and developing assistive technologies.

## Limitations

Our study had several limitations that may have impacted the outcomes and their interpretation. First, for behavioral measures of lipreading skill, we predominantly focused on higher-level comprehension, i.e., we examined participants’ skill using only continuous text rather than isolated words or sentences, likely overlooking critical aspects of lipreading at more basic levels. This oversight may have prevented us from capturing important variations in lipreading abilities among participants who may or may not struggle with fundamental skills.

Another limitation was that we used random order of experimental conditions and for a few participants we firstly performed tasks with auditory before attempting them without sound. Although this sequence was unnoticed by most due to the overall difficulty of the tasks, it could introduce variability in the results, decreasing the power of fMRI analysis.

Moreover, we did not provide any lipreading training before the experiment to familiarize participants with the specificity of lipreading. We did not investigate the linguistic capabilities of the individuals involved, which might have influenced their performance in lipreading tasks. Future studies should focus on those two aspects to possibly reduce the variability of strategies used by participants and therefore decrease the variance in behavioral and neurocognitive strategies used during lipreading.

## Electronic supplementary material

Below is the link to the electronic supplementary material.

Supplementary Material 1

## Data Availability

Behavioral data and code used for statistical analysis is available at OSF repository: (https://colab.research.google.com/drive/1nJWiWisgWB_Uyu4Bt0sdDJldPDaeteVw?usp=sharing). Unthresholded, group-level whole-brain neuroimaging results maps are available at public repository Neurovault: (https://osf.io/6k74t/). Raw neuroimaging data are not available due to the privacy regulations.
