# Supplementary material for: Neural mechanisms of lipreading in the Polish-speaking population: effects of linguistic complexity and sex differences
Source: Sci Rep. 2025 Apr 17;15:13253. doi: 10.1038/s41598-025-98026-8 (PMC12006354; doi:10.1038/s41598-025-98026-8)
Supplement: Supplementary file 1 — Supplementary Material 1 [file 41598_2025_98026_MOESM1_ESM.docx]

**Title: Neural mechanisms of lipreading in the Polish-speaking population: effects of linguistic complexity and sex differences**

Authors: Jakub Wojciechowski, Joanna Beck, Hanna Cygan, Agnieszka Pankowska, Tomasz Wolak

Supplementary materials

Table of content

[With voice conditions: speech sentences vs words 2](#_Toc178860145)

[Conjunction analysis for 'visual lexical vs. face' with 'audiovisual lexical vs. face 3](#_Toc178860146)

[Table S1. Audiovisual sentences > Audiovisual words. Figure S1 4](#_Toc178860147)

[Table S2. Audiovisual words > Audiovisual sentences. Figure S1 5](#_Toc178860148)

[Table S3. Visual lexical sentences > words. Figure 6 6](#_Toc178860149)

[Table S4. Visual lexical words > sentences. Figure 6 7](#_Toc178860150)

[Table S5. Visual non-lexical sentences > words. Figure 6 8](#_Toc178860151)

[Table S6. Visual non-lexical words > sentences. Figure 6 8](#_Toc178860152)

[Table S7. Visual lexical sentences > face. Figure 4 9](#_Toc178860153)

[Table S8. Face > Visual lexical sentences. Figure 4 10](#_Toc178860154)

[Table S9. Visual lexical sentences > audiovisual. Figure 4 11](#_Toc178860155)

[Table S10. Audiovisual lexical sentences > visual. Figure 4 11](#_Toc178860156)

[Table S11. Visual lexical sentences > non-lexical. Figure 4 12](#_Toc178860157)

[Table S12. Visual non-lexical sentences > lexical. Figure 4 13](#_Toc178860158)

[Table. S13. Visual lexical words > face. Figure 5 13](#_Toc178860159)

[Table. S14. Face > Visual lexical words. Figure 5 14](#_Toc178860160)

[Table S15. Visual lexical words > audiovisual words. Figure 5 15](#_Toc178860161)

[Table S16. Audiovisual words > visual lexical words. Figure 5 15](#_Toc178860162)

[Table S17. Visual lexical words > non-lexical. Figure 5 17](#_Toc178860163)

[Table S18. Visual non-lexical words > lexical. Figure S1 17](#_Toc178860164)

[Table S19. Conjunction of visual lexical vs face ∧ audiovisual vs face. Figure S2 18](#_Toc178860165)

[Table S20. Conjunction of face vs visual lexical ∧ face vs audiovisual. Figure S2 19](#_Toc178860166)

#

# With voice conditions: speech sentences vs words

We observed the involvement of dorsal and ventral neural pathways typically involved in language processing. When processing sentences several brain regions showed heightened activity i.e., bilateral middle and superior temporal areas as well as Heschl sulcus (including posterior speech area. Wernicke’s), bilateral medial superior and orbito-frontal areas (interior speech area. Broca’s area), bilateral precuneus, bilateral cingulate gyrus, right superior occipital gyrus and right cerebellum.
When processing individual words there was higher activation in the bilateral orbitofrontal gyrus, bilateral medial superior frontal areas, bilateral inferior and superior parietal lobule, bilateral superior occipital area, left inferior and middle occipital cortex.


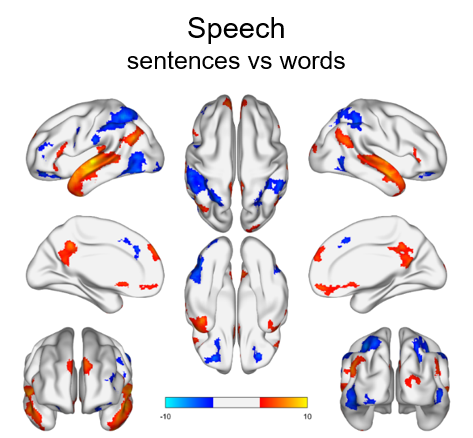


Figure S1. Brain map activations for audiovisual sentences vs words comparisons. Contrast maps are thresholded at voxel‐level *p* < .001 and FWE‐corrected (*p* < .05) for cluster size.

#

# Conjunction analysis for 'visual lexical vs. face' with 'audiovisual lexical vs. Face

We observed that both only visual and audiovisual lexical processing engaged language and visual brain network: bilateral middle and superior temporal cortex, bilateral frontal and middle superior frontal areas (supplementary motor areas) and bilateral occipital cortex.


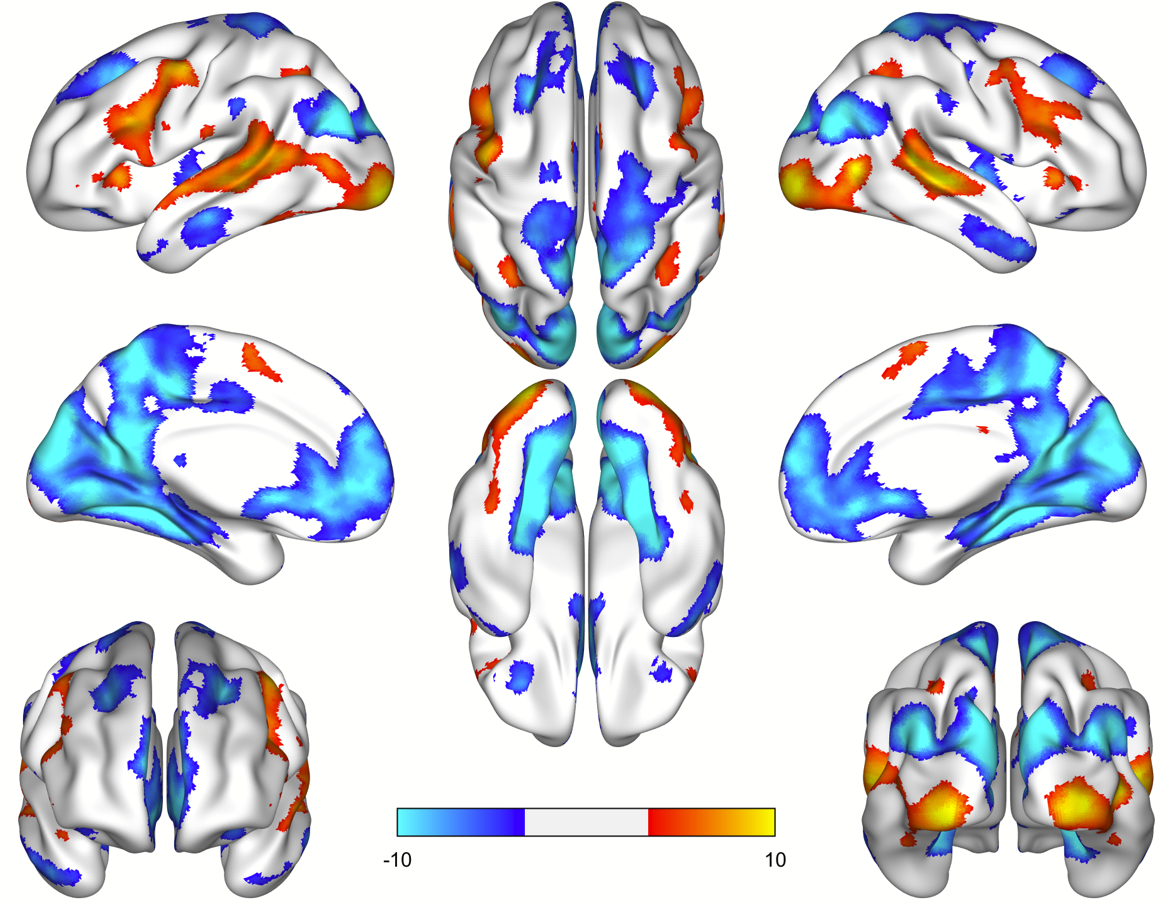


Figure S2. Brain map activations for conjunction of visual lexical vs face and audiovisual vs face comparisons. Contrast maps are thresholded at voxel‐level *p* < .001 and FWE‐corrected (*p* < .05) for cluster size.

In all of the tables functional regions are reported with corresponding MNI coordinates of peak activity within each cluster as well as cluster size. Additionally noted is the percentage of cluster's voxel in each anatomical region defined using the AAL3 atlas. Only regions with more than 5% of all cluster's voxels are noted. Results are thresholded at p<0.001 for voxel and FWE-corrected (p<0.05) for cluster size.

For the sake of clarity, two tables are presented for each contrast, listing only the t-values that are significantly greater in one condition vs. the other.

Unthresholded group-level whole-brain results maps are available at Neurovault repository: <https://neurovault.org/collections/TZJFBYKF/>

#

# Table S1. Audiovisual sentences > Audiovisual words. Figure S1

| Coordinates MNI | | | Cluster size | Region (% of cluster) | Cluster | | | Peak |
| --- | --- | --- | --- | --- | --- | --- | --- | --- |
|  |  |  |  |  | pFWE | pFDR | p(uncor) | T |
| z | y | z |  |  |  |  |  |  |
| -62 | -16 | -4 | 5921 | (39%) Temporal_Mid_L | <0.001 | <0.001 | <0.001 | 10.76 |
|  |  |  |  | (16%) Temporal_Sup_L |  |  |  |  |
|  |  |  |  | (10%) Angular_L |  |  |  |  |
|  |  |  |  | (8%) OUTSIDE |  |  |  |  |
|  |  |  |  | (6%) Temporal_Pole_Sup_L |  |  |  |  |
|  |  |  |  | (6%) Temporal_Pole_Mid_L |  |  |  |  |
|  |  |  |  | (6%) Temporal_Inf_L |  |  |  |  |
| 64 | -12 | 2 | 3605 | (38%) Temporal_Sup_R | <0.001 | <0.001 | <0.001 | 9.55 |
|  |  |  |  | (28%) Temporal_Mid_R |  |  |  |  |
|  |  |  |  | (14%) Temporal_Pole_Sup_R |  |  |  |  |
|  |  |  |  | (10%) Temporal_Pole_Mid_R |  |  |  |  |
| 20 | -82 | -36 | 911 | (47%) Cerebelum_Crus1_R | <0.001 | <0.001 | <0.001 | 7.92 |
|  |  |  |  | (39%) Cerebelum_Crus2_R |  |  |  |  |
|  |  |  |  | (8%) Cerebelum_6_R |  |  |  |  |
| -8 | -48 | 40 | 1483 | (33%) Precuneus_L | <0.001 | <0.001 | <0.001 | 7.79 |
|  |  |  |  | (24%) Precuneus_R |  |  |  |  |
|  |  |  |  | (11%) Cingulate_Mid_R |  |  |  |  |
|  |  |  |  | (10%) Cingulate_Post_L |  |  |  |  |
|  |  |  |  | (8%) Cingulate_Mid_L |  |  |  |  |
|  |  |  |  | (5%) Cingulate_Post_R |  |  |  |  |
| 52 | 28 | -2 | 155 | (88%) Frontal_Inf_Tri_R | 0.013 | 0.002 | 0.001 | 6.81 |
|  |  |  |  | (12%) Frontal_Inf_Orb_2_R |  |  |  |  |
| 48 | -54 | 22 | 858 | (62%) Angular_R | <0.001 | <0.001 | <0.001 | 6.71 |
|  |  |  |  | (21%) Temporal_Mid_R |  |  |  |  |
|  |  |  |  | (7%) Temporal_Sup_R |  |  |  |  |
|  |  |  |  | (6%) OUTSIDE |  |  |  |  |
| 6 | -52 | -42 | 420 | (49%) Cerebelum_9_R | <0.001 | <0.001 | <0.001 | 6.38 |
|  |  |  |  | (35%) Cerebelum_9_L |  |  |  |  |
|  |  |  |  | (8%) Vermis_9 |  |  |  |  |
|  |  |  |  | (7%) OUTSIDE |  |  |  |  |
| -8 | 54 | 36 | 960 | (42%) Frontal_Sup_Medial_L | <0.001 | <0.001 | <0.001 | 6.3 |
|  |  |  |  | (41%) Frontal_Sup_Medial_R |  |  |  |  |
|  |  |  |  | (11%) Frontal_Sup_2_L |  |  |  |  |
| -12 | -86 | -38 | 391 | (72%) Cerebelum_Crus2_L | <0.001 | <0.001 | <0.001 | 6.03 |
|  |  |  |  | (27%) Cerebelum_Crus1_L |  |  |  |  |
| 2 | 54 | -8 | 641 | (35%) Frontal_Med_Orb_R | <0.001 | <0.001 | <0.001 | 5.62 |
|  |  |  |  | (28%) Frontal_Med_Orb_L |  |  |  |  |
|  |  |  |  | (10%) Olfactory_L |  |  |  |  |
|  |  |  |  | (9%) Rectus_R |  |  |  |  |
|  |  |  |  | (5%) Olfactory_R |  |  |  |  |
| -48 | 26 | 8 | 236 | (97%) Frontal_Inf_Tri_L | 0.001 | <0.001 | <0.001 | 5.17 |
| 20 | -98 | 20 | 183 | (50%) Occipital_Sup_R | 0.005 | 0.001 | <0.001 | 4.99 |
|  |  |  |  | (21%) Cuneus_R |  |  |  |  |
|  |  |  |  | (20%) Occipital_Mid_R |  |  |  |  |
|  |  |  |  | (9%) OUTSIDE |  |  |  |  |

# Table S2. Audiovisual words > Audiovisual sentences. Figure S1

| Coordinates MNI | | | Cluster size | Region (% of cluster) | Cluster | | | Peak |
| --- | --- | --- | --- | --- | --- | --- | --- | --- |
|  |  |  |  |  | pFWE | pFDR | p(uncor) | T |
| z | y | z |  |  |  |  |  |  |
| -32 | -50 | 52 | 2546 | (52%) Parietal_Inf_L | <0.001 | <0.001 | <0.001 | 7.11 |
|  |  |  |  | (25%) Parietal_Sup_L |  |  |  |  |
|  |  |  |  | (16%) Postcentral_L |  |  |  |  |
| -50 | -68 | -8 | 1154 | (31%) Temporal_Inf_L | <0.001 | <0.001 | <0.001 | 6.6 |
|  |  |  |  | (22%) Temporal_Mid_L |  |  |  |  |
|  |  |  |  | (22%) Occipital_Inf_L |  |  |  |  |
|  |  |  |  | (16%) Occipital_Mid_L |  |  |  |  |
| 2 | 20 | 44 | 499 | (37%) Frontal_Sup_Medial_L | <0.001 | <0.001 | <0.001 | 6.38 |
|  |  |  |  | (28%) Supp_Motor_Area_L |  |  |  |  |
|  |  |  |  | (10%) Cingulate_Mid_R |  |  |  |  |
|  |  |  |  | (7%) Supp_Motor_Area_R |  |  |  |  |
|  |  |  |  | (6%) ACC_sup_L |  |  |  |  |
|  |  |  |  | (6%) Frontal_Sup_Medial_R |  |  |  |  |
|  |  |  |  | (5%) Cingulate_Mid_L |  |  |  |  |
| -26 | 46 | -14 | 293 | (46%) OFCant_L | <0.001 | <0.001 | <0.001 | 6.3 |
|  |  |  |  | (16%) OFCpost_L |  |  |  |  |
|  |  |  |  | (13%) OFClat_L |  |  |  |  |
|  |  |  |  | (10%) OFCmed_L |  |  |  |  |
|  |  |  |  | (10%) Frontal_Mid_2_L |  |  |  |  |
| 26 | 42 | -14 | 284 | (43%) OFCant_R | <0.001 | <0.001 | <0.001 | 6.19 |
|  |  |  |  | (34%) Frontal_Mid_2_R |  |  |  |  |
|  |  |  |  | (8%) OFCmed_R |  |  |  |  |
|  |  |  |  | (8%) Frontal_Sup_2_R |  |  |  |  |
|  |  |  |  | (5%) OUTSIDE |  |  |  |  |
| 34 | -94 | -6 | 126 | (96%) Occipital_Inf_R | 0.034 | 0.006 | 0.001 | 6.17 |
| 46 | -40 | 52 | 1651 | (39%) Parietal_Inf_R | <0.001 | <0.001 | <0.001 | 6.12 |
|  |  |  |  | (27%) Parietal_Sup_R |  |  |  |  |
|  |  |  |  | (8%) Postcentral_R |  |  |  |  |
|  |  |  |  | (8%) SupraMarginal_R |  |  |  |  |
|  |  |  |  | (8%) Occipital_Sup_R |  |  |  |  |
|  |  |  |  | (8%) OUTSIDE |  |  |  |  |
| 48 | -60 | -14 | 393 | (48%) Temporal_Inf_R | <0.001 | <0.001 | <0.001 | 5.82 |
|  |  |  |  | (46%) Temporal_Mid_R |  |  |  |  |
| -42 | 2 | 32 | 237 | (86%) Precentral_L | 0.001 | <0.001 | <0.001 | 5.35 |
|  |  |  |  | (12%) Frontal_Inf_Oper_L |  |  |  |  |
| -28 | -96 | -12 | 148 | (40%) Occipital_Inf_L | 0.016 | 0.003 | 0.001 | 5.11 |
|  |  |  |  | (28%) OUTSIDE |  |  |  |  |
|  |  |  |  | (24%) Occipital_Mid_L |  |  |  |  |
|  |  |  |  | (7%) Lingual_L |  |  |  |  |
| -36 | 42 | 12 | 335 | (54%) Frontal_Mid_2_L | <0.001 | <0.001 | <0.001 | 4.8 |
|  |  |  |  | (46%) Frontal_Inf_Tri_L |  |  |  |  |

# Table S3. Visual lexical sentences > words. Figure 6

| Coordinates MNI | | | Cluster size | Region (% of cluster) | Cluster | | | Peak |
| --- | --- | --- | --- | --- | --- | --- | --- | --- |
|  |  |  |  |  | pFWE | pFDR | p(uncor) | T |
| z | y | z |  |  |  |  |  |  |
| -2 | -38 | 44 | 2511 | (37%) Precuneus_L | <0.001 | <0.001 | <0.001 | 6.7 |
|  |  |  |  | (22%) Precuneus_R |  |  |  |  |
|  |  |  |  | (13%) Cingulate_Mid_L |  |  |  |  |
|  |  |  |  | (11%) Cingulate_Mid_R |  |  |  |  |
|  |  |  |  | (8%) Cingulate_Post_L |  |  |  |  |
|  |  |  |  | (5%) Cingulate_Post_R |  |  |  |  |
| 2 | 60 | 18 | 1371 | (34%) Frontal_Sup_Medial_L | <0.001 | <0.001 | <0.001 | 6.49 |
|  |  |  |  | (30%) Frontal_Sup_Medial_R | |  |  |  |
|  |  |  |  | (12%) ACC_pre_L |  |  |  |  |
|  |  |  |  | (8%) ACC_pre_R |  |  |  |  |
|  |  |  |  | (7%) Frontal_Med_Orb_R |  |  |  |  |
|  |  |  |  | (5%) Frontal_Med_Orb_L |  |  |  |  |
| -42 | -62 | 28 | 564 | (83%) Angular_L | <0.001 | <0.001 | <0.001 | 5.28 |
|  |  |  |  | (7%) Occipital_Mid_L |  |  |  |  |
| -54 | -18 | -28 | 390 | (68%) Temporal_Mid_L | <0.001 | <0.001 | <0.001 | 5.18 |
|  |  |  |  | (32%) Temporal_Inf_L |  |  |  |  |
| 48 | -62 | 32 | 176 | (81%) Angular_R | 0.005 | 0.003 | <0.001 | 4.42 |
|  |  |  |  | (13%) Temporal_Mid_R |  |  |  |  |

# Table S4. Visual lexical words > sentences. Figure 6

| Coordinates MNI | | | Cluster size | Region (% of cluster) | Cluster | | | Peak |
| --- | --- | --- | --- | --- | --- | --- | --- | --- |
|  |  |  |  |  | pFWE | pFDR | p(uncor) | T |
| z | y | z |  |  |  |  |  |  |
| -44 | 2 | 32 | 2181 | (46%) Precentral_L | <0.001 | <0.001 | <0.001 | 7.64 |
|  |  |  |  | (25%) Frontal_Inf_Oper_L |  |  |  |  |
|  |  |  |  | (15%) Frontal_Inf_Tri_L |  |  |  |  |
| -58 | -36 | 16 | 1003 | (32%) Temporal_Sup_L | <0.001 | <0.001 | <0.001 | 6.79 |
|  |  |  |  | (19%) SupraMarginal_L |  |  |  |  |
|  |  |  |  | (18%) Postcentral_L |  |  |  |  |
|  |  |  |  | (15%) Parietal_Inf_L |  |  |  |  |
|  |  |  |  | (13%) Temporal_Mid_L |  |  |  |  |
| 50 | 0 | 52 | 1003 | (42%) Precentral_R | <0.001 | <0.001 | <0.001 | 6.47 |
|  |  |  |  | (37%) Frontal_Inf_Oper_R |  |  |  |  |
|  |  |  |  | (15%) Frontal_Mid_2_R |  |  |  |  |
| 52 | -62 | -6 | 514 | (56%) Temporal_Inf_R | <0.001 | <0.001 | <0.001 | 6.1 |
|  |  |  |  | (35%) Temporal_Mid_R |  |  |  |  |
|  |  |  |  | (7%) Occipital_Inf_R |  |  |  |  |
| -36 | 24 | 6 | 132 | (62%) Insula_L | 0.022 | 0.005 | 0.001 | 6.07 |
|  |  |  |  | (38%) Frontal_Inf_Tri_L |  |  |  |  |
| 64 | -16 | 24 | 720 | (38%) SupraMarginal_R | <0.001 | <0.001 | <0.001 | 5.93 |
|  |  |  |  | (35%) Postcentral_R |  |  |  |  |
|  |  |  |  | (21%) Temporal_Sup_R |  |  |  |  |
| -44 | -70 | -10 | 571 | (37%) Temporal_Mid_L | <0.001 | <0.001 | <0.001 | 5.73 |
|  |  |  |  | (21%) Occipital_Inf_L |  |  |  |  |
|  |  |  |  | (21%) Occipital_Mid_L |  |  |  |  |
|  |  |  |  | (12%) Temporal_Inf_L |  |  |  |  |
|  |  |  |  | (6%) Fusiform_L |  |  |  |  |
| 28 | -52 | 56 | 143 | (66%) Parietal_Sup_R | 0.015 | 0.004 | 0.001 | 5.21 |
|  |  |  |  | (31%) Parietal_Inf_R |  |  |  |  |
| 18 | -72 | -46 | 130 | (65%) Cerebelum_8_R | 0.024 | 0.005 | 0.001 | 5.13 |
|  |  |  |  | (30%) Cerebelum_7b_R |  |  |  |  |
|  |  |  |  | (5%) Cerebelum_Crus2_R |  |  |  |  |
| 52 | 26 | 18 | 147 | (87%) Frontal_Inf_Tri_R | 0.013 | 0.004 | 0.001 | 4.31 |
|  |  |  |  | (13%) Frontal_Mid_2_R |  |  |  |  |
| 36 | -38 | 56 | 142 | (99%) Postcentral_R | 0.016 | 0.004 | 0.001 | 4.28 |

# Table S5. Visual non-lexical sentences > words. Figure 6

| Coordinates MNI | | | Cluster size | Region (% of cluster) | Cluster | | | Peak |
| --- | --- | --- | --- | --- | --- | --- | --- | --- |
|  |  |  |  |  | pFWE | pFDR | p(uncor) | T |
| z | y | z |  |  |  |  |  |  |
| -8 | -46 | 34 | 2093 | (38%) Precuneus_L | <0.001 | <0.001 | <0.001 | 7.95 |
|  |  |  |  | (20%) Precuneus_R |  |  |  |  |
|  |  |  |  | (10%) Cingulate_Mid_L | |  |  |  |
|  |  |  |  | (9%) Cingulate_Post_L | |  |  |  |
|  |  |  |  | (8%) Cingulate_Mid_R | |  |  |  |
|  |  |  |  | (8%) OUTSIDE |  |  |  |  |
|  |  |  |  | (4%) Cingulate_Post_R | |  |  |  |
| -50 | -58 | 24 | 1507 | (57%) Angular_L | <0.001 | <0.001 | <0.001 | 7.92 |
|  |  |  |  | (13%) Parietal_Inf_L |  |  |  |  |
|  |  |  |  | (12%) OUTSIDE |  |  |  |  |
|  |  |  |  | (8%) Temporal_Mid_L | |  |  |  |
|  |  |  |  | (6%) SupraMarginal_L | |  |  |  |
| -60 | 6 | -22 | 790 | (68%) Temporal_Mid_L | <0.001 | <0.001 | <0.001 | 5.63 |
|  |  |  |  | (23%) Temporal_Inf_L | |  |  |  |
|  |  |  |  | (5%) Temporal_Pole_Mid_L | |  |  |  |
| -26 | -84 | -32 | 158 | (56%) Cerebelum_Crus2_L | 0.011 | 0.004 | <0.001 | 5.56 |
|  |  |  |  | (42%) Cerebelum_Crus1_L | |  |  |  |
| 18 | -88 | -32 | 547 | (49%) Cerebelum_Crus2_R | <0.001 | <0.001 | <0.001 | 5.56 |
|  |  |  |  | (48%) Cerebelum_Crus1_R | |  |  |  |
| 46 | -56 | 24 | 508 | (83%) Angular_R | <0.001 | <0.001 | <0.001 | 5.46 |
|  |  |  |  | (6%) Temporal_Mid_R | |  |  |  |
|  |  |  |  | (6%) Occipital_Mid_R |  |  |  |  |
| 2 | -78 | -6 | 125 | (49%) Lingual_L | 0.034 | 0.012 | 0.001 | 5.46 |
|  |  |  |  | (28%) Lingual_R |  |  |  |  |
|  |  |  |  | (12%) Calcarine_L |  |  |  |  |
|  |  |  |  | (9%) OUTSIDE |  |  |  |  |
| -38 | 10 | 48 | 164 | (91%) Frontal_Mid_2_L | 0.009 | 0.004 | <0.001 | 4.39 |

#

# Table S6. Visual non-lexical words > sentences. Figure 6

| Coordinates MNI | | | Cluster size | Region (% of cluster) | Cluster | | | Peak |
| --- | --- | --- | --- | --- | --- | --- | --- | --- |
|  |  |  |  |  | pFWE | pFDR | p(uncor) | T |
| z | y | z |  |  |  |  |  |  |
| -48 | 6 | 28 | 3405 | (27%) Precentral_L | <0.001 | <0.001 | <0.001 | 7.4 |
|  |  |  |  | (25%) Postcentral_L |  |  |  |  |
|  |  |  |  | (11%) Frontal_Inf_Oper_L |  |  |  |  |
|  |  |  |  | (10%) Temporal_Sup_L |  |  |  |  |
|  |  |  |  | (9%) Parietal_Inf_L |  |  |  |  |
|  |  |  |  | (6%) SupraMarginal_L |  |  |  |  |
| -36 | 20 | 2 | 163 | (86%) Insula_L | 0.01 | 0.002 | <0.001 | 5.89 |
|  |  |  |  | (13%) Frontal_Inf_Tri_L |  |  |  |  |
| 48 | 10 | 24 | 512 | (47%) Precentral_R | <0.001 | <0.001 | <0.001 | 5.6 |
|  |  |  |  | (34%) Frontal_Inf_Oper_R |  |  |  |  |
|  |  |  |  | (16%) Frontal_Mid_2_R |  |  |  |  |
| 0 | 10 | 50 | 379 | (70%) Supp_Motor_Area_L | <0.001 | <0.001 | <0.001 | 5.43 |
|  |  |  |  | (18%) Supp_Motor_Area_R |  |  |  |  |
|  |  |  |  | (5%) Cingulate_Mid_L |  |  |  |  |
|  |  |  |  | (5%) Frontal_Sup_Medial_L |  |  |  |  |
| -52 | -62 | -8 | 676 | (29%) Temporal_Mid_L | <0.001 | <0.001 | <0.001 | 5.39 |
|  |  |  |  | (27%) Temporal_Inf_L |  |  |  |  |
|  |  |  |  | (17%) Occipital_Inf_L |  |  |  |  |
|  |  |  |  | (14%) Fusiform_L |  |  |  |  |
|  |  |  |  | (9%) Occipital_Mid_L |  |  |  |  |
| 50 | -60 | 4 | 282 | (77%) Temporal_Mid_R | <0.001 | <0.001 | <0.001 | 5.22 |
|  |  |  |  | (20%) Temporal_Inf_R |  |  |  |  |
| -44 | 34 | 14 | 204 | (85%) Frontal_Inf_Tri_L | 0.003 | 0.001 | <0.001 | 5.2 |
|  |  |  |  | (14%) Frontal_Mid_2_L |  |  |  |  |
| 32 | -50 | 62 | 644 | (54%) Postcentral_R | <0.001 | <0.001 | <0.001 | 5.04 |
|  |  |  |  | (31%) Parietal_Sup_R |  |  |  |  |
|  |  |  |  | (8%) Parietal_Inf_R |  |  |  |  |
| 56 | -22 | 16 | 173 | (55%) Temporal_Sup_R | 0.007 | 0.002 | <0.001 | 4.58 |
|  |  |  |  | (17%) SupraMarginal_R |  |  |  |  |
|  |  |  |  | (14%) Postcentral_R |  |  |  |  |
|  |  |  |  | (14%) Rolandic_Oper_R |  |  |  |  |

#

# Table S7. Visual lexical sentences > face. Figure 4

| Coordinates MNI | | | Cluster size | Region (% of cluster) | Cluster | | | Peak |
| --- | --- | --- | --- | --- | --- | --- | --- | --- |
|  |  |  |  |  | pFWE | pFDR | p(uncor) | T |
| z | y | z |  |  |  |  |  |  |
| 0 | 2 | 66 | 2411 | (41%) Supp_Motor_Area_L | <0.001 | <0.001 | <0.001 | 14.68 |
|  |  |  |  | (28%) Supp_Motor_Area_R |  |  |  |  |
|  |  |  |  | (12%) Frontal_Sup_Medial_L |  |  |  |  |
|  |  |  |  | (9%) Cingulate_Mid_R |  |  |  |  |
|  |  |  |  | (6%) Frontal_Sup_Medial_R |  |  |  |  |
| -30 | 24 | 2 | 30832 | (7%) Frontal_Mid_2_R | <0.001 | <0.001 | <0.001 | 14.5 |
|  |  |  |  | (6%) Temporal_Mid_L |  |  |  |  |
|  |  |  |  | (5%) OUTSIDE |  |  |  |  |
|  |  |  |  | (5%) Precentral_L |  |  |  |  |
|  |  |  |  | (5%) Frontal_Inf_Tri_L |  |  |  |  |
| -38 | -54 | 56 | 1846 | (60%) Parietal_Inf_L | <0.001 | <0.001 | <0.001 | 9.86 |
|  |  |  |  | (27%) Parietal_Sup_L |  |  |  |  |
|  |  |  |  | (8%) Postcentral_L |  |  |  |  |
| -18 | 2 | 4 | 1031 | (26%) OUTSIDE | <0.001 | <0.001 | <0.001 | 8.76 |
|  |  |  |  | (17%) Putamen_L |  |  |  |  |
|  |  |  |  | (13%) Caudate_R |  |  |  |  |
|  |  |  |  | (12%) Caudate_L |  |  |  |  |
|  |  |  |  | (9%) Pallidum_L |  |  |  |  |
|  |  |  |  | (6%) Pallidum_R |  |  |  |  |
|  |  |  |  | (5%) Thal_VL_L |  |  |  |  |
| 0 | -30 | 26 | 265 | (75%) OUTSIDE | 0.001 | <0.001 | <0.001 | 7.8 |
|  |  |  |  | (18%) Cingulate_Mid_R |  |  |  |  |

# Table S8. Face > Visual lexical sentences. Figure 4

| Coordinates MNI | | | Cluster size | Region (% of cluster) | Cluster | | | Peak |
| --- | --- | --- | --- | --- | --- | --- | --- | --- |
|  |  |  |  |  | pFWE | pFDR | p(uncor) | T |
| z | y | z |  |  |  |  |  |  |
| 14 | -90 | 16 | 54527 | (17%) OUTSIDE | <0.001 | <0.001 | <0.001 | 17.79 |
|  |  |  |  | (5%) Precuneus_L |  |  |  |  |
| -14 | -52 | -48 | 630 | (43%) Cerebelum_9_L | <0.001 | <0.001 | <0.001 | 8.11 |
|  |  |  |  | (40%) Cerebelum_8_L |  |  |  |  |
|  |  |  |  | (9%) Cerebelum_Crus1_L |  |  |  |  |
| -30 | 36 | -12 | 257 | (43%) OFCpost_L | 0.001 | <0.001 | <0.001 | 7.84 |
|  |  |  |  | (28%) Frontal_Inf_Orb_2_L |  |  |  |  |
|  |  |  |  | (15%) OFCant_L |  |  |  |  |
|  |  |  |  | (10%) Frontal_Mid_2_L |  |  |  |  |
|  |  |  |  | (4%) OFClat_L |  |  |  |  |
| -30 | -82 | -32 | 288 | (52%) Cerebelum_Crus1_L | 0.001 | <0.001 | <0.001 | 6.87 |
|  |  |  |  | (48%) Cerebelum_Crus2_L |  |  |  |  |

# Table S9. Visual lexical sentences > audiovisual. Figure 4

| Coordinates MNI | | | Cluster size | Region (% of cluster) | Cluster | | | Peak |
| --- | --- | --- | --- | --- | --- | --- | --- | --- |
|  |  |  |  |  | pFWE | pFDR | p(uncor) | T |
| z | y | z |  |  |  |  |  |  |
| 4 | 22 | 44 | 26469 | (12%) Frontal_Mid_2_R | <0.001 | <0.001 | <0.001 | 13.89 |
|  |  |  |  | (11%) OUTSIDE |  |  |  |  |
|  |  |  |  | (10%) Frontal_Mid_2_L |  |  |  |  |
|  |  |  |  | (7%) Frontal_Sup_2_R |  |  |  |  |
|  |  |  |  | (7%) Frontal_Sup_2_L |  |  |  |  |
|  |  |  |  | (6%) Precentral_L |  |  |  |  |
|  |  |  |  | (5%) Supp_Motor_Area_L |  |  |  |  |
| 48 | -66 | -10 | 10064 | (13%) OUTSIDE | <0.001 | <0.001 | <0.001 | 11.01 |
|  |  |  |  | (11%) Parietal_Sup_R |  |  |  |  |
|  |  |  |  | (10%) Parietal_Inf_R |  |  |  |  |
|  |  |  |  | (9%) Occipital_Mid_R |  |  |  |  |
|  |  |  |  | (8%) Occipital_Inf_R |  |  |  |  |
|  |  |  |  | (7%) Temporal_Mid_R |  |  |  |  |
|  |  |  |  | (6%) Temporal_Inf_R |  |  |  |  |
|  |  |  |  | (5%) SupraMarginal_R |  |  |  |  |
|  |  |  |  | (5%) Precuneus_R |  |  |  |  |
| -46 | -70 | 2 | 9818 | (18%) Parietal_Inf_L | <0.001 | <0.001 | <0.001 | 10.46 |
|  |  |  |  | (16%) Occipital_Mid_L |  |  |  |  |
|  |  |  |  | (11%) Parietal_Sup_L |  |  |  |  |
|  |  |  |  | (10%) OUTSIDE |  |  |  |  |
|  |  |  |  | (7%) Occipital_Inf_L |  |  |  |  |
|  |  |  |  | (7%) Cerebelum_Crus1_L |  |  |  |  |

# Table S10. Audiovisual lexical sentences > visual. Figure 4

| Coordinates MNI | | | Cluster size | Region (% of cluster) | Cluster | | | Peak |
| --- | --- | --- | --- | --- | --- | --- | --- | --- |
|  |  |  |  |  | pFWE | pFDR | p(uncor) | T |
| z | y | z |  |  |  |  |  |  |
| -48 | -18 | 8 | 8763 | (25%) Temporal_Mid_L | <0.001 | <0.001 | <0.001 | 25.51 |
|  |  |  |  | (25%) Temporal_Sup_L |  |  |  |  |
|  |  |  |  | (10%) OUTSIDE |  |  |  |  |
|  |  |  |  | (8%) Temporal_Pole_Sup_L |  |  |  |  |
|  |  |  |  | (7%) Rolandic_Oper_L |  |  |  |  |
|  |  |  |  | (7%) Angular_L |  |  |  |  |
| 48 | -18 | 8 | 14253 | (17%) Temporal_Sup_R | <0.001 | <0.001 | <0.001 | 21.75 |
|  |  |  |  | (11%) Calcarine_L |  |  |  |  |
|  |  |  |  | (9%) Temporal_Mid_R |  |  |  |  |
|  |  |  |  | (8%) Calcarine_R |  |  |  |  |
|  |  |  |  | (6%) OUTSIDE |  |  |  |  |
|  |  |  |  | (5%) Temporal_Pole_Sup_R |  |  |  |  |
|  |  |  |  | (5%) Rolandic_Oper_R |  |  |  |  |
| -4 | 48 | -12 | 1124 | (32%) Frontal_Med_Orb_L | <0.001 | <0.001 | <0.001 | 8.85 |
|  |  |  |  | (20%) Rectus_L |  |  |  |  |
|  |  |  |  | (18%) Frontal_Med_Orb_R |  |  |  |  |
|  |  |  |  | (11%) Rectus_R |  |  |  |  |
|  |  |  |  | (7%) Olfactory_L |  |  |  |  |
|  |  |  |  | (5%) OUTSIDE |  |  |  |  |
| -16 | -28 | -4 | 155 | (34%) OUTSIDE | 0.012 | 0.002 | <0.001 | 8.25 |
|  |  |  |  | (14%) Hippocampus_L |  |  |  |  |
|  |  |  |  | (14%) Thal_PuM_L |  |  |  |  |
|  |  |  |  | (10%) Thal_PuI_L |  |  |  |  |
|  |  |  |  | (8%) Thal_MGN_L |  |  |  |  |
|  |  |  |  | (8%) Thal_LGN_L |  |  |  |  |
|  |  |  |  | (7%) Thal_VPL_L |  |  |  |  |
| 50 | -62 | 26 | 685 | (64%) Angular_R | <0.001 | <0.001 | <0.001 | 8.23 |
|  |  |  |  | (22%) Temporal_Mid_R |  |  |  |  |
|  |  |  |  | (7%) Occipital_Mid_R |  |  |  |  |
|  |  |  |  | (6%) Temporal_Sup_R |  |  |  |  |
| -6 | 60 | 26 | 987 | (55%) Frontal_Sup_Medial_L | <0.001 | <0.001 | <0.001 | 6.96 |
|  |  |  |  | (30%) Frontal_Sup_Medial_R |  |  |  |  |
|  |  |  |  | (10%) Frontal_Sup_2_L |  |  |  |  |
| 22 | -84 | -34 | 240 | (56%) Cerebelum_Crus1_R | 0.001 | <0.001 | <0.001 | 5.58 |
|  |  |  |  | (44%) Cerebelum_Crus2_R |  |  |  |  |

# Table S11. Visual lexical sentences > non-lexical. Figure 4

| Coordinates MNI | | | Cluster size | Region (% of cluster) | Cluster | | | Peak |
| --- | --- | --- | --- | --- | --- | --- | --- | --- |
|  |  |  |  |  | pFWE | pFDR | p(uncor) | T |
| z | y | z |  |  |  |  |  |  |
| 0 | -2 | 68 | 735 | (81%) Supp_Motor_Area_L | <0.001 | <0.001 | <0.001 | 8.59 |
|  |  |  |  | (19%) Supp_Motor_Area_R |  |  |  |  |
| -58 | -26 | -2 | 4773 | (25%) Temporal_Mid_L | <0.001 | <0.001 | <0.001 | 7.21 |
|  |  |  |  | (16%) Frontal_Inf_Tri_L |  |  |  |  |
|  |  |  |  | (15%) Precentral_L |  |  |  |  |
|  |  |  |  | (11%) Temporal_Sup_L |  |  |  |  |
|  |  |  |  | (9%) Frontal_Inf_Oper_L |  |  |  |  |
|  |  |  |  | (6%) Temporal_Pole_Sup_L |  |  |  |  |
|  |  |  |  | (5%) Frontal_Inf_Orb_2_L |  |  |  |  |
| 12 | -82 | -46 | 556 | (44%) Cerebelum_Crus2_R | <0.001 | <0.001 | <0.001 | 5.74 |
|  |  |  |  | (22%) Cerebelum_8_R |  |  |  |  |
|  |  |  |  | (13%) Cerebelum_Crus1_R |  |  |  |  |
|  |  |  |  | (12%) Cerebelum_7b_R |  |  |  |  |
|  |  |  |  | (9%) Cerebelum_6_R |  |  |  |  |
| 30 | -60 | -22 | 142 | (92%) Cerebelum_6_R | 0.016 | 0.003 | 0.001 | 5.28 |
|  |  |  |  | (6%) Fusiform_R |  |  |  |  |
| 60 | 8 | -14 | 193 | (74%) Temporal_Pole_Sup_R | 0.003 | 0.001 | <0.001 | 5.1 |
|  |  |  |  | (20%) Temporal_Sup_R |  |  |  |  |
| 2 | -18 | 12 | 261 | (54%) OUTSIDE | <0.001 | <0.001 | <0.001 | 4.95 |
|  |  |  |  | (19%) Caudate_L |  |  |  |  |
|  |  |  |  | (8%) Putamen_L |  |  |  |  |
|  |  |  |  | (8%) Pallidum_L |  |  |  |  |
| 50 | -28 | -2 | 146 | (64%) Temporal_Sup_R | 0.014 | 0.003 | 0.001 | 4.27 |
|  |  |  |  | (28%) Temporal_Mid_R |  |  |  |  |
|  |  |  |  | (8%) OUTSIDE |  |  |  |  |

# Table S12. Visual non-lexical sentences > lexical. Figure 4

| Coordinates MNI | | | Cluster size | Region (% of cluster) | Cluster | | | Peak |
| --- | --- | --- | --- | --- | --- | --- | --- | --- |
|  |  |  |  |  | pFWE | pFDR | p(uncor) | T |
| z | y | z |  |  |  |  |  |  |
| 50 | -54 | 32 | 219 | (80%) Angular_R | 0.001 | 0.002 | <0.001 | 5.8 |
|  |  |  |  | (11%) Parietal_Inf_R |  |  |  |  |
|  |  |  |  | (9%) SupraMarginal_R |  |  |  |  |
| 10 | 48 | 0 | 483 | (54%) Frontal_Sup_Medial_R | <0.001 | <0.001 | <0.001 | 5.68 |
|  |  |  |  | (16%) ACC_pre_R |  |  |  |  |
|  |  |  |  | (11%) Frontal_Sup_Medial_L |  |  |  |  |
|  |  |  |  | (8%) ACC_pre_L |  |  |  |  |
|  |  |  |  | (7%) Frontal_Med_Orb_R |  |  |  |  |

# Table. S13. Visual lexical words > face. Figure 5

| Coordinates MNI | | | Cluster size | Region (% of cluster) | Cluster | | | Peak |
| --- | --- | --- | --- | --- | --- | --- | --- | --- |
|  |  |  |  |  | pFWE | pFDR | p(uncor) | T |
| z | y | z |  |  |  |  |  |  |
| 0 | 2 | 64 | 2599 | (37%) Supp_Motor_Area_L | <0.001 | <0.001 | <0.001 | 16.92 |
|  |  |  |  | (25%) Supp_Motor_Area_R |  |  |  |  |
|  |  |  |  | (13%) Frontal_Sup_Medial_L |  |  |  |  |
|  |  |  |  | (10%) Cingulate_Mid_R |  |  |  |  |
|  |  |  |  | (5%) Cingulate_Mid_L |  |  |  |  |
|  |  |  |  | (5%) Frontal_Sup_Medial_R |  |  |  |  |
| -30 | 22 | 2 | 28696 | (9%) Frontal_Mid_2_R | <0.001 | <0.001 | <0.001 | 15.78 |
|  |  |  |  | (6%) Precentral_L |  |  |  |  |
|  |  |  |  | (6%) Temporal_Mid_L |  |  |  |  |
|  |  |  |  | (5%) Frontal_Inf_Tri_L |  |  |  |  |
|  |  |  |  | (5%) Temporal_Mid_R |  |  |  |  |
| -22 | -2 | 6 | 887 | (28%) Putamen_L | <0.001 | <0.001 | <0.001 | 11.08 |
|  |  |  |  | (23%) Caudate_L |  |  |  |  |
|  |  |  |  | (21%) OUTSIDE |  |  |  |  |
|  |  |  |  | (11%) Pallidum_L |  |  |  |  |
|  |  |  |  | (7%) Thal_VL_L |  |  |  |  |
| -36 | -56 | 56 | 1632 | (57%) Parietal_Inf_L | <0.001 | <0.001 | <0.001 | 9.63 |
|  |  |  |  | (30%) Parietal_Sup_L |  |  |  |  |
|  |  |  |  | (8%) Postcentral_L |  |  |  |  |
| 44 | -46 | 56 | 2178 | (45%) Parietal_Inf_R | <0.001 | <0.001 | <0.001 | 9.25 |
|  |  |  |  | (27%) Parietal_Sup_R |  |  |  |  |
|  |  |  |  | (9%) Angular_R |  |  |  |  |
|  |  |  |  | (7%) SupraMarginal_R |  |  |  |  |
|  |  |  |  | (6%) Postcentral_R |  |  |  |  |
| 22 | 0 | 4 | 772 | (23%) OUTSIDE | <0.001 | <0.001 | <0.001 | 8.92 |
|  |  |  |  | (23%) Caudate_R |  |  |  |  |
|  |  |  |  | (16%) Putamen_R |  |  |  |  |
|  |  |  |  | (12%) Pallidum_R |  |  |  |  |
|  |  |  |  | (10%) Thal_VL_R |  |  |  |  |
| 2 | -28 | 26 | 226 | (75%) OUTSIDE | 0.003 | <0.001 | <0.001 | 7.65 |
|  |  |  |  | (22%) Cingulate_Mid_R |  |  |  |  |

# Table. S14. Face > Visual lexical words. Figure 5

| Coordinates MNI | | | Cluster size | Region (% of cluster) | Cluster | | | Peak |
| --- | --- | --- | --- | --- | --- | --- | --- | --- |
|  |  |  |  |  | pFWE | pFDR | p(uncor) | T |
| z | y | z |  |  |  |  |  |  |
| -26 | -50 | -10 | 72544 | (30%) OUTSIDE | <0.001 | <0.001 | <0.001 | 15.37 |
| 12 | -48 | -48 | 2512 | (26%) OUTSIDE | <0.001 | <0.001 | <0.001 | 9.12 |
|  |  |  |  | (17%) Cerebelum_Crus1_R |  |  |  |  |
|  |  |  |  | (15%) Cerebelum_9_L |  |  |  |  |
|  |  |  |  | (13%) Cerebelum_9_R |  |  |  |  |
|  |  |  |  | (12%) Cerebelum_8_L |  |  |  |  |
|  |  |  |  | (12%) Cerebelum_Crus2_R |  |  |  |  |
| -30 | -82 | -34 | 370 | (56%) Cerebelum_Crus2_L | <0.001 | <0.001 | <0.001 | 7.67 |
|  |  |  |  | (43%) Cerebelum_Crus1_L |  |  |  |  |

# Table S15. Visual lexical words > audiovisual words. Figure 5

| Coordinates MNI | | | Cluster size | Region (% of cluster) | Cluster | | | Peak |
| --- | --- | --- | --- | --- | --- | --- | --- | --- |
|  |  |  |  |  | pFWE | pFDR | p(uncor) | T |
| z | y | z |  |  |  |  |  |  |
| -4 | 4 | 60 | 28361 | (10%) OUTSIDE | <0.001 | <0.001 | <0.001 | 13.42 |
|  |  |  |  | (9%) Frontal_Mid_2_R |  |  |  |  |
|  |  |  |  | (6%) Frontal_Inf_Tri_L |  |  |  |  |
|  |  |  |  | (6%) Precentral_L |  |  |  |  |
|  |  |  |  | (5%) Frontal_Mid_2_L |  |  |  |  |
| 46 | -62 | 6 | 11182 | (11%) Occipital_Mid_L | <0.001 | <0.001 | <0.001 | 10.68 |
|  |  |  |  | (9%) OUTSIDE |  |  |  |  |
|  |  |  |  | (9%) Cerebelum_6_R |  |  |  |  |
|  |  |  |  | (7%) Occipital_Inf_R |  |  |  |  |
|  |  |  |  | (6%) Temporal_Mid_R |  |  |  |  |
|  |  |  |  | (6%) Occipital_Mid_R |  |  |  |  |
|  |  |  |  | (5%) Cerebelum_6_L |  |  |  |  |
|  |  |  |  | (5%) Occipital_Inf_L |  |  |  |  |
|  |  |  |  | (5%) Cerebelum_Crus1_L |  |  |  |  |
|  |  |  |  | (5%) Temporal_Inf_R |  |  |  |  |
| 34 | -52 | 60 | 2479 | (25%) Parietal_Inf_R | <0.001 | <0.001 | <0.001 | 6.31 |
|  |  |  |  | (21%) Parietal_Sup_R |  |  |  |  |
|  |  |  |  | (21%) Postcentral_R |  |  |  |  |
|  |  |  |  | (13%) SupraMarginal_R |  |  |  |  |
|  |  |  |  | (11%) OUTSIDE |  |  |  |  |
| -4 | -22 | 28 | 222 | (75%) OUTSIDE | 0.003 | 0.001 | <0.001 | 5.75 |
|  |  |  |  | (19%) Cingulate_Mid_R |  |  |  |  |
|  |  |  |  | (6%) Cingulate_Mid_L |  |  |  |  |
| 14 | -64 | 40 | 137 | (91%) Precuneus_R | 0.03 | 0.007 | 0.001 | 4.68 |
|  |  |  |  | (7%) Cuneus_R |  |  |  |  |

# Table S16. Audiovisual words > visual lexical words. Figure 5

| Coordinates MNI | | | Cluster size | Region (% of cluster) | Cluster | | | Peak |
| --- | --- | --- | --- | --- | --- | --- | --- | --- |
|  |  |  |  |  | pFWE | pFDR | p(uncor) | T |
| z | y | z |  |  |  |  |  |  |
| 50 | -16 | 6 | 5169 | (43%) Temporal_Sup_R | <0.001 | <0.001 | <0.001 | 22.16 |
|  |  |  |  | (14%) Temporal_Mid_R |  |  |  |  |
|  |  |  |  | (13%) Rolandic_Oper_R |  |  |  |  |
|  |  |  |  | (7%) Insula_R |  |  |  |  |
|  |  |  |  | (7%) Temporal_Pole_Sup_R |  |  |  |  |
|  |  |  |  | (5%) OUTSIDE |  |  |  |  |
|  |  |  |  | (5%) Heschl_R |  |  |  |  |
| -50 | -16 | 6 | 5116 | (38%) Temporal_Sup_L | <0.001 | <0.001 | <0.001 | 21.81 |
|  |  |  |  | (26%) Temporal_Mid_L |  |  |  |  |
|  |  |  |  | (10%) Rolandic_Oper_L |  |  |  |  |
|  |  |  |  | (10%) OUTSIDE |  |  |  |  |
| 12 | -96 | 20 | 6509 | (17%) Calcarine_L | <0.001 | <0.001 | <0.001 | 8.02 |
|  |  |  |  | (13%) Precuneus_L |  |  |  |  |
|  |  |  |  | (12%) Calcarine_R |  |  |  |  |
|  |  |  |  | (9%) Cuneus_L |  |  |  |  |
|  |  |  |  | (8%) Precuneus_R |  |  |  |  |
|  |  |  |  | (8%) Cuneus_R |  |  |  |  |
|  |  |  |  | (7%) Lingual_R |  |  |  |  |
|  |  |  |  | (7%) Lingual_L |  |  |  |  |
| -50 | -62 | 24 | 1331 | (66%) Angular_L | <0.001 | <0.001 | <0.001 | 7.7 |
|  |  |  |  | (9%) Temporal_Mid_L |  |  |  |  |
|  |  |  |  | (9%) Occipital_Mid_L |  |  |  |  |
|  |  |  |  | (8%) OUTSIDE |  |  |  |  |
|  |  |  |  | (7%) Parietal_Inf_L |  |  |  |  |
| 0 | 40 | -22 | 550 | (44%) Frontal_Med_Orb_L | <0.001 | <0.001 | <0.001 | 6.25 |
|  |  |  |  | (32%) Rectus_L |  |  |  |  |
|  |  |  |  | (16%) Rectus_R |  |  |  |  |
|  |  |  |  | (5%) Frontal_Med_Orb_R |  |  |  |  |
| 46 | -64 | 32 | 464 | (82%) Angular_R | <0.001 | <0.001 | <0.001 | 5.78 |
|  |  |  |  | (8%) Temporal_Mid_R |  |  |  |  |
| -10 | 62 | 28 | 325 | (68%) Frontal_Sup_Medial_L | <0.001 | <0.001 | <0.001 | 5.29 |
|  |  |  |  | (21%) Frontal_Sup_Medial_R |  |  |  |  |
|  |  |  |  | (11%) Frontal_Sup_2_L |  |  |  |  |
| -18 | 40 | 46 | 170 | (82%) Frontal_Sup_2_L | 0.011 | 0.002 | <0.001 | 4.46 |
|  |  |  |  | (14%) Frontal_Sup_Medial_L |  |  |  |  |

# Table S17. Visual lexical words > non-lexical. Figure 5

| Coordinates MNI | | | Cluster size | Region (% of cluster) | Cluster | | | Peak |
| --- | --- | --- | --- | --- | --- | --- | --- | --- |
|  |  |  |  |  | pFWE | pFDR | p(uncor) | T |
| z | y | z |  |  |  |  |  |  |
| -2 | 2 | 58 | 1156 | (55%) Supp_Motor_Area_L | <0.001 | <0.001 | <0.001 | 7.3 |
|  |  |  |  | (12%) Supp_Motor_Area_R |  |  |  |  |
|  |  |  |  | (11%) Cingulate_Mid_L |  |  |  |  |
|  |  |  |  | (8%) Cingulate_Mid_R |  |  |  |  |
|  |  |  |  | (6%) Frontal_Sup_Medial_L |  |  |  |  |
| -32 | 16 | 8 | 1823 | (25%) Temporal_Sup_L | <0.001 | <0.001 | <0.001 | 6.18 |
|  |  |  |  | (22%) Temporal_Mid_L |  |  |  |  |
|  |  |  |  | (16%) Insula_L |  |  |  |  |
|  |  |  |  | (12%) Temporal_Pole_Sup_L |  |  |  |  |
|  |  |  |  | (6%) Frontal_Inf_Orb_2_L |  |  |  |  |
|  |  |  |  | (5%) Frontal_Inf_Oper_L |  |  |  |  |
| -14 | 2 | 14 | 583 | (36%) OUTSIDE | <0.001 | <0.001 | <0.001 | 6.01 |
|  |  |  |  | (26%) Putamen_L |  |  |  |  |
|  |  |  |  | (17%) Caudate_L |  |  |  |  |
|  |  |  |  | (7%) Pallidum_L |  |  |  |  |
| -46 | 10 | 32 | 365 | (41%) Precentral_L | <0.001 | <0.001 | <0.001 | 5.51 |
|  |  |  |  | (32%) Frontal_Inf_Tri_L |  |  |  |  |
|  |  |  |  | (15%) Frontal_Inf_Oper_L |  |  |  |  |
|  |  |  |  | (13%) Frontal_Mid_2_L |  |  |  |  |
| -38 | -6 | 62 | 409 | (67%) Precentral_L | <0.001 | <0.001 | <0.001 | 5.22 |
|  |  |  |  | (18%) Frontal_Sup_2_L |  |  |  |  |
|  |  |  |  | (12%) Postcentral_L |  |  |  |  |
| 18 | 16 | -2 | 220 | (44%) Putamen_R | 0.002 | <0.001 | <0.001 | 5.2 |
|  |  |  |  | (29%) Caudate_R |  |  |  |  |
|  |  |  |  | (25%) OUTSIDE |  |  |  |  |
| -32 | 50 | 20 | 304 | (86%) Frontal_Mid_2_L | <0.001 | <0.001 | <0.001 | 5.07 |
|  |  |  |  | (8%) Frontal_Sup_2_L |  |  |  |  |
|  |  |  |  | (6%) Frontal_Inf_Tri_L |  |  |  |  |
| 40 | 18 | -4 | 130 | (93%) Insula_R | 0.033 | 0.007 | 0.001 | 4.87 |
| 32 | 46 | 36 | 262 | (97%) Frontal_Mid_2_R | 0.001 | <0.001 | <0.001 | 4.61 |

# Table S18. Visual non-lexical words > lexical. Figure S1

| Coordinates MNI | | | Cluster size | Region (% of cluster) | Cluster | | | Peak |
| --- | --- | --- | --- | --- | --- | --- | --- | --- |
|  |  |  |  |  | pFWE | pFDR | p(uncor) | T |
| z | y | z |  |  |  |  |  |  |
| 48 | -52 | 26 | 419 | (61%) Angular_R | <0.001 | <0.001 | <0.001 | 6.44 |
|  |  |  |  | (16%) SupraMarginal_R |  |  |  |  |
|  |  |  |  | (10%) OUTSIDE |  |  |  |  |
|  |  |  |  | (8%) Temporal_Mid_R |  |  |  |  |
| -22 | -76 | -34 | 400 | (59%) Cerebelum_Crus2_L | <0.001 | <0.001 | <0.001 | 6.28 |
|  |  |  |  | (41%) Cerebelum_Crus1_L |  |  |  |  |
| 38 | -12 | 18 | 219 | (57%) Insula_R | 0.002 | 0.002 | <0.001 | 6.18 |
|  |  |  |  | (38%) Rolandic_Oper_R |  |  |  |  |
| 34 | -50 | -10 | 132 | (80%) Fusiform_R | 0.031 | 0.017 | 0.001 | 5.03 |
|  |  |  |  | (10%) Lingual_R |  |  |  |  |
|  |  |  |  | (9%) OUTSIDE |  |  |  |  |
| 68 | -20 | 36 | 245 | (77%) SupraMarginal_R | 0.001 | 0.001 | <0.001 | 4.98 |
|  |  |  |  | (11%) Postcentral_R |  |  |  |  |
|  |  |  |  | (9%) OUTSIDE |  |  |  |  |
| 40 | -80 | 22 | 144 | (100%) Occipital_Mid_R | 0.021 | 0.014 | 0.001 | 4.18 |

# Table S19. Conjunction of visual lexical vs face ∧ audiovisual vs face. Figure S2

| Coordinates MNI | | | Cluster size | Region (% of cluster) | Cluster | Cluster | Cluster | Peak |
| --- | --- | --- | --- | --- | --- | --- | --- | --- |
|  |  |  |  |  | pFWE | pFDR | p(uncor) | T |
| z | y | z |  |  |  |  |  |  |
| -28 | -98 | -8 | 12879 | (13%) Temporal_Mid_L | < 0.001 | < 0.001 | < 0.001 | 13.51 |
|  |  |  |  | (9%) Precentral_L |  |  |  |  |
|  |  |  |  | (8%) Temporal_Sup_L |  |  |  |  |
|  |  |  |  | (8%) Frontal_Inf_Tri_L |  |  |  |  |
|  |  |  |  | (6%) OUTSIDE |  |  |  |  |
|  |  |  |  | (6%) Occipital_Inf_R |  |  |  |  |
| 56 | -24 | -2 | 5584 | (22%) Temporal_Sup_R | < 0.001 | < 0.001 | < 0.001 | 11.37 |
|  |  |  |  | (15%) Frontal_Mid_2_R |  |  |  |  |
|  |  |  |  | (15%) Frontal_Inf_Tri_R |  |  |  |  |
|  |  |  |  | (15%) Temporal_Mid_R |  |  |  |  |
|  |  |  |  | (9%) Frontal_Inf_Oper_R |  |  |  |  |
|  |  |  |  | (8%) Precentral_R |  |  |  |  |
|  |  |  |  | (5%) Temporal_Pole_Sup_R |  |  |  |  |
| 0 | 0 | 64 | 794 | (59%) Supp_Motor_Area_L | < 0.001 | < 0.001 | < 0.001 | 10.49 |
|  |  |  |  | (40%) Supp_Motor_Area_R |  |  |  |  |
| -36 | -58 | 58 | 662 | (61%) Parietal_Inf_L | < 0.001 | < 0.001 | < 0.001 | 7.54 |
|  |  |  |  | (35%) Parietal_Sup_L |  |  |  |  |
| 40 | -52 | 62 | 900 | (48%) Parietal_Inf_R | < 0.001 | < 0.001 | < 0.001 | 7.04 |
|  |  |  |  | (34%) Parietal_Sup_R |  |  |  |  |
|  |  |  |  | (17%) Angular_R |  |  |  |  |
|  |  |  |  |  |  |  |  |  |

# Table S20. Conjunction of face vs visual lexical ∧ face vs audiovisual. Figure S2

| Coordinates MNI | | | Cluster size | Region (% of cluster) | Cluster | Cluster | Cluster | Peak |
| --- | --- | --- | --- | --- | --- | --- | --- | --- |
|  |  |  |  |  | pFWE | pFDR | p(uncor) | T |
| z | y | z |  |  |  |  |  |  |
| 28 | -42 | -8 | 70023 | (30%) OUTSIDE | < 0.001 | < 0.001 | < 0.001 | 16.9 |
| -32 | -82 | -32 | 268 | (58%) Cerebelum_Crus1_L | 0.001 | < 0.001 | < 0.001 | 8.16 |
|  |  |  |  | (42%) Cerebelum_Crus2_L |  |  |  |  |
